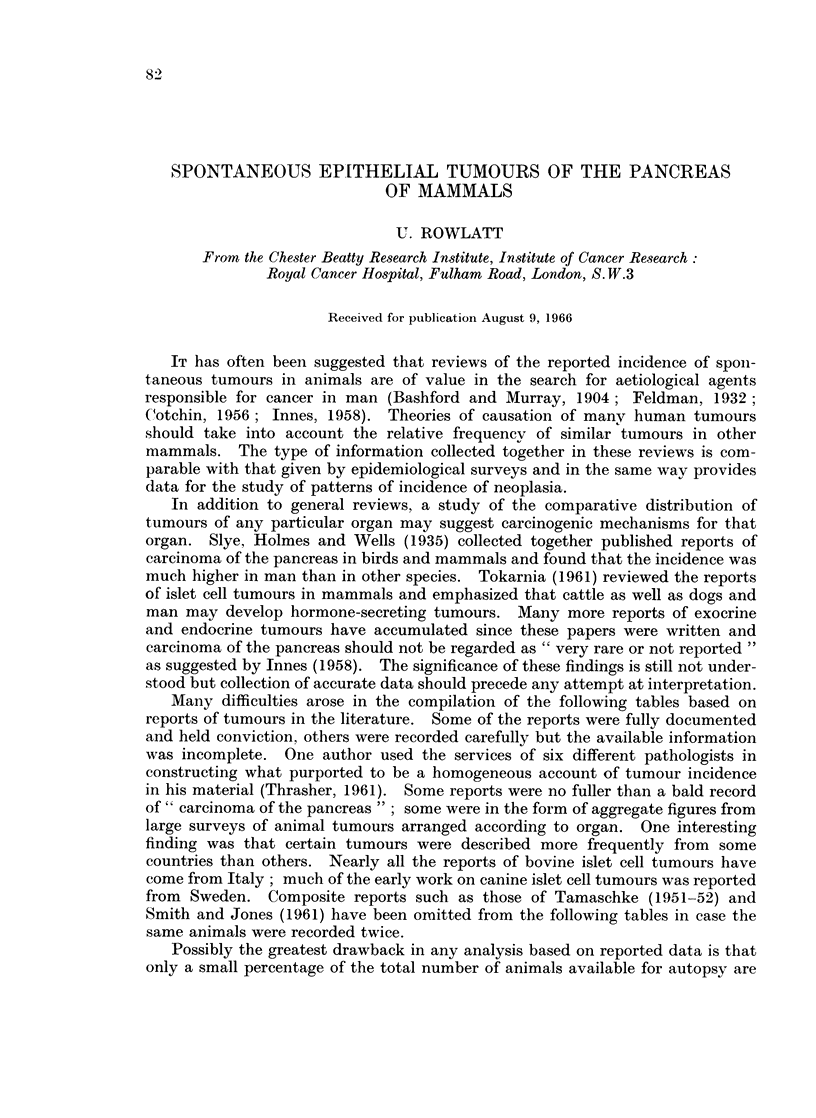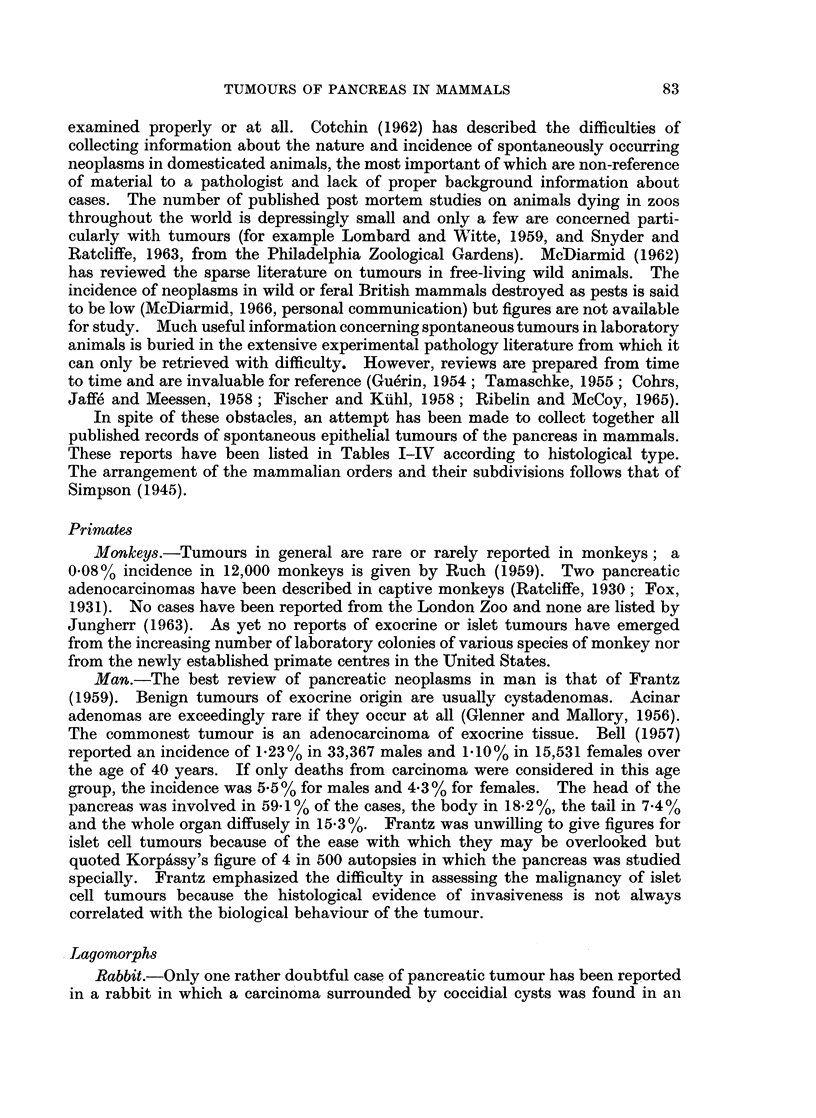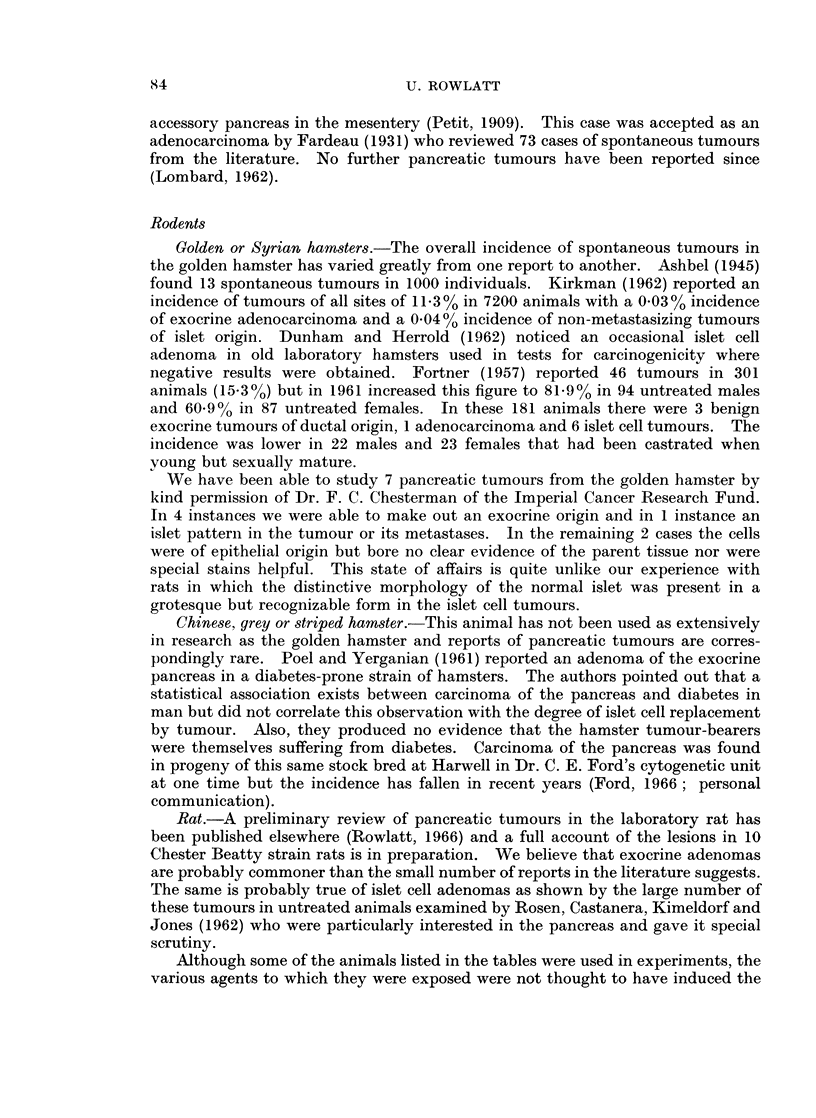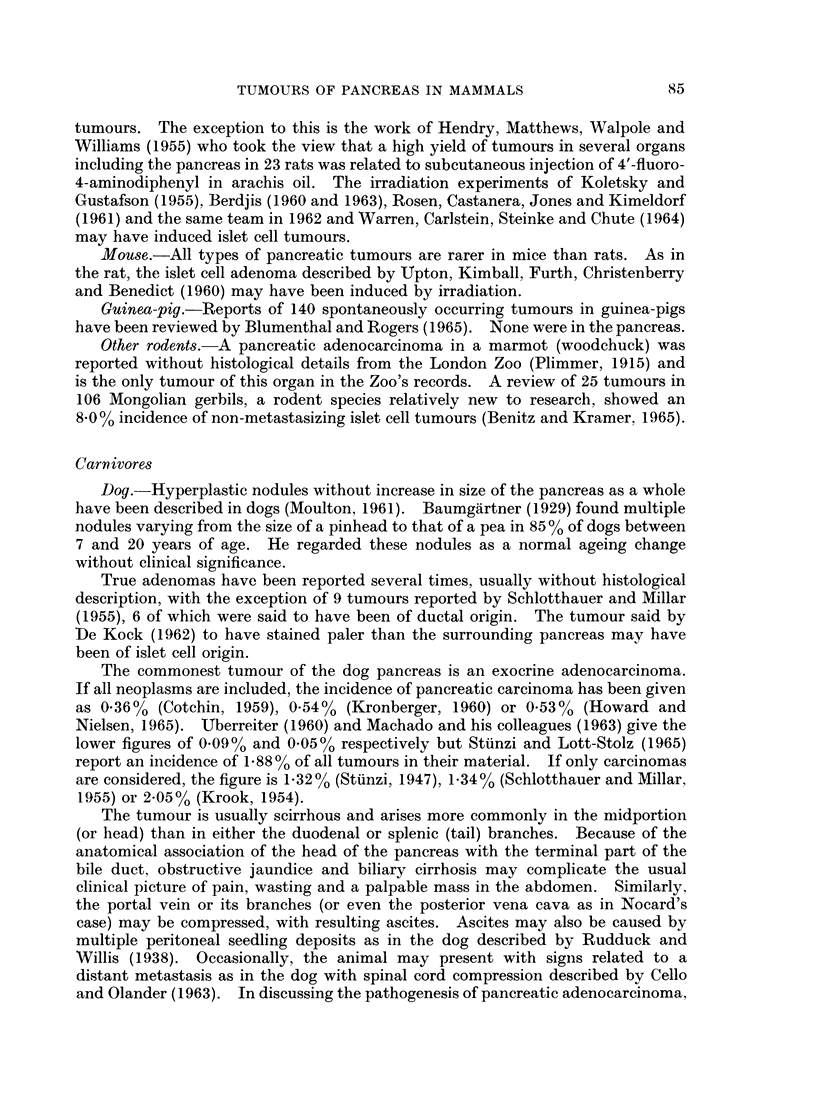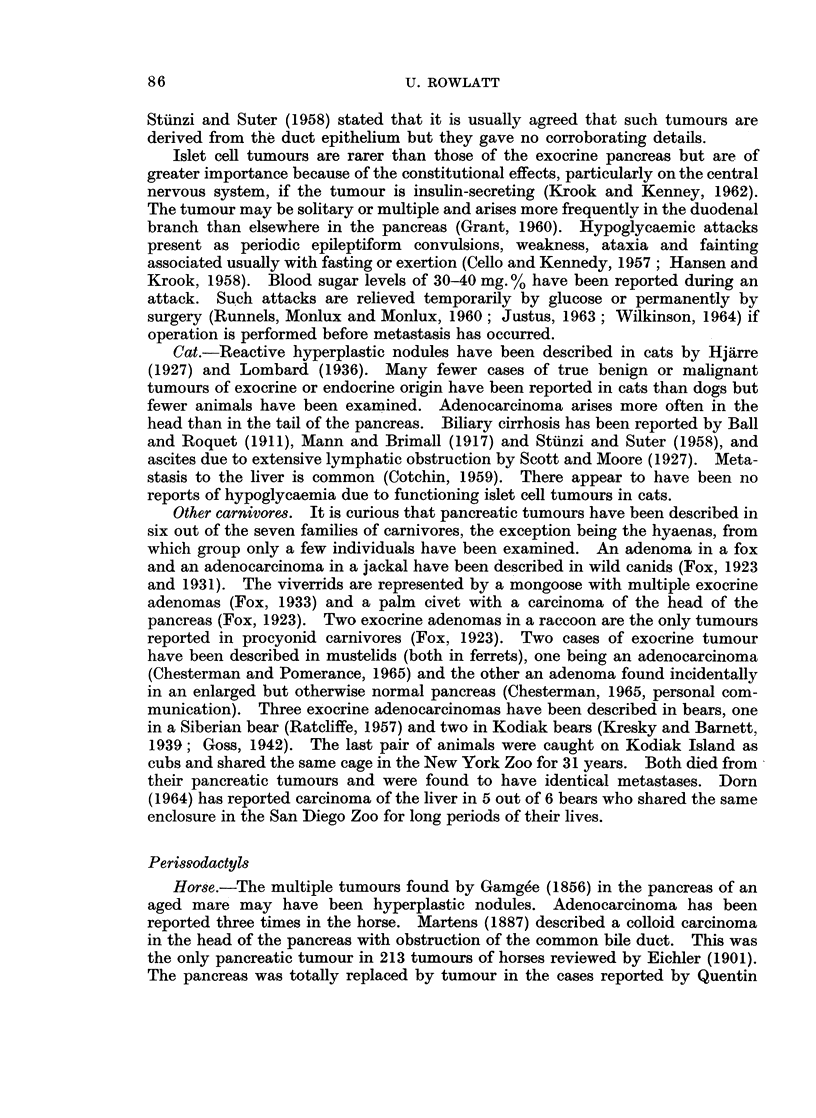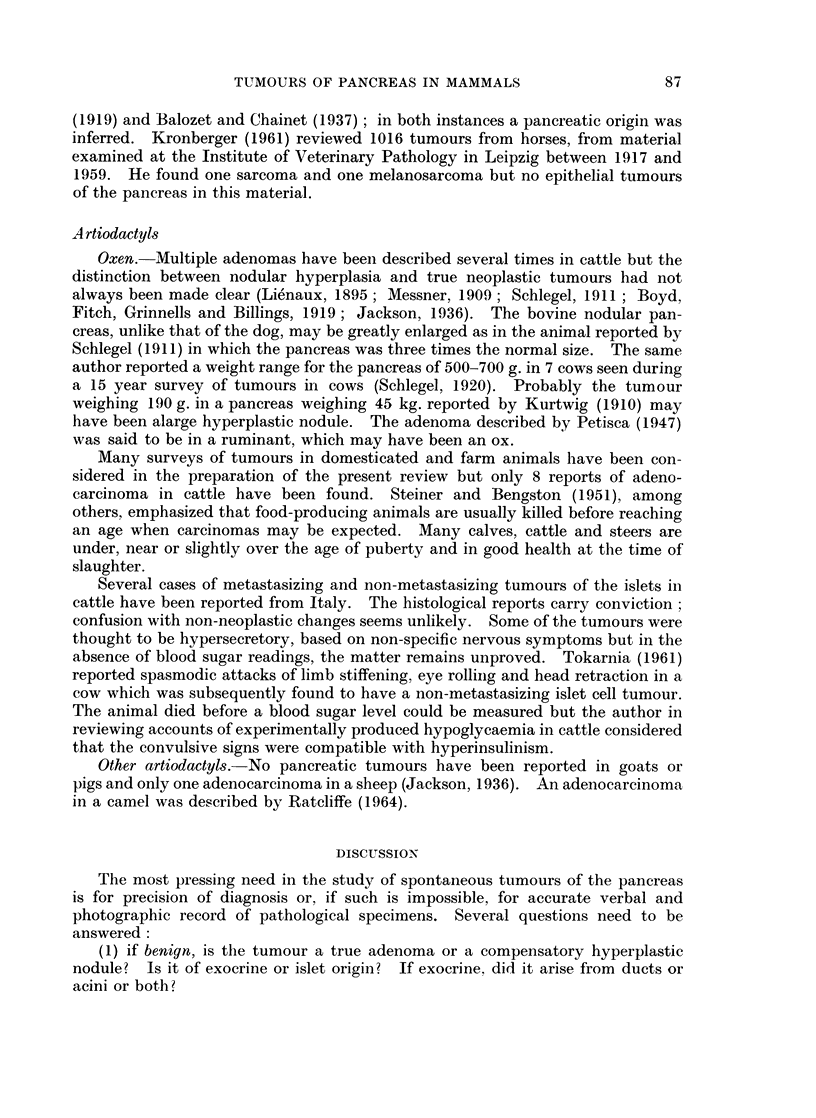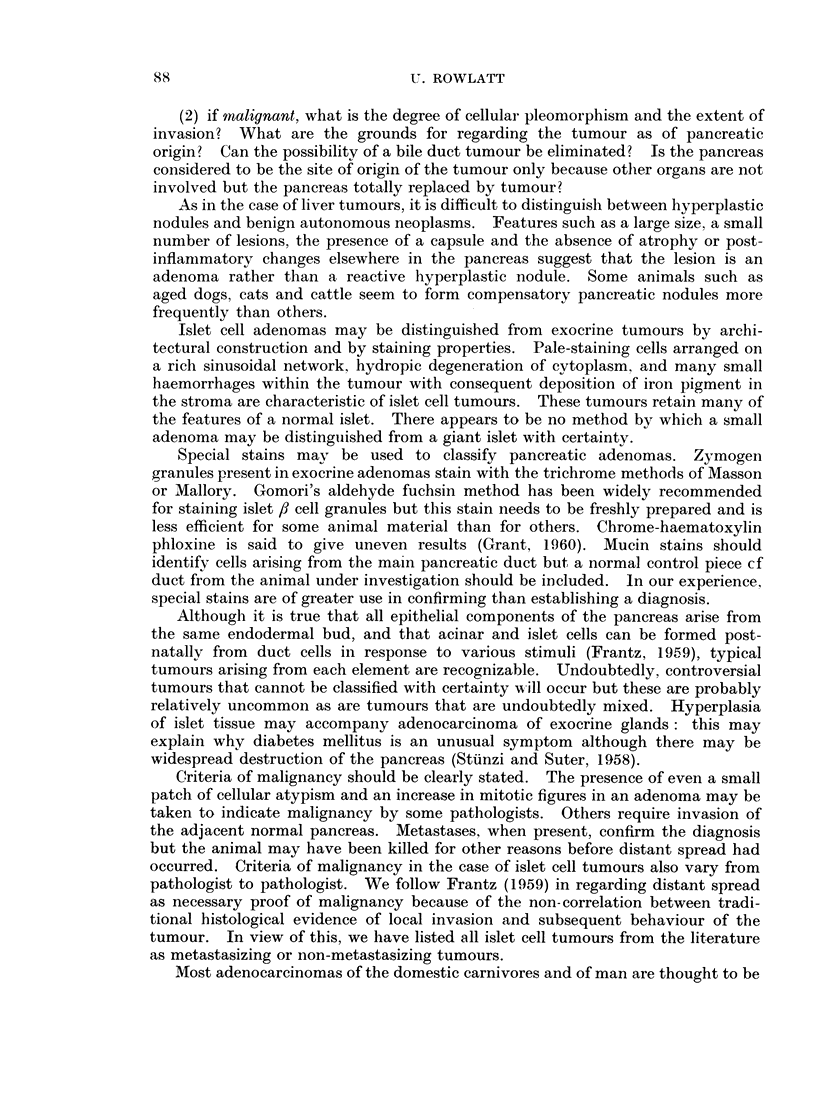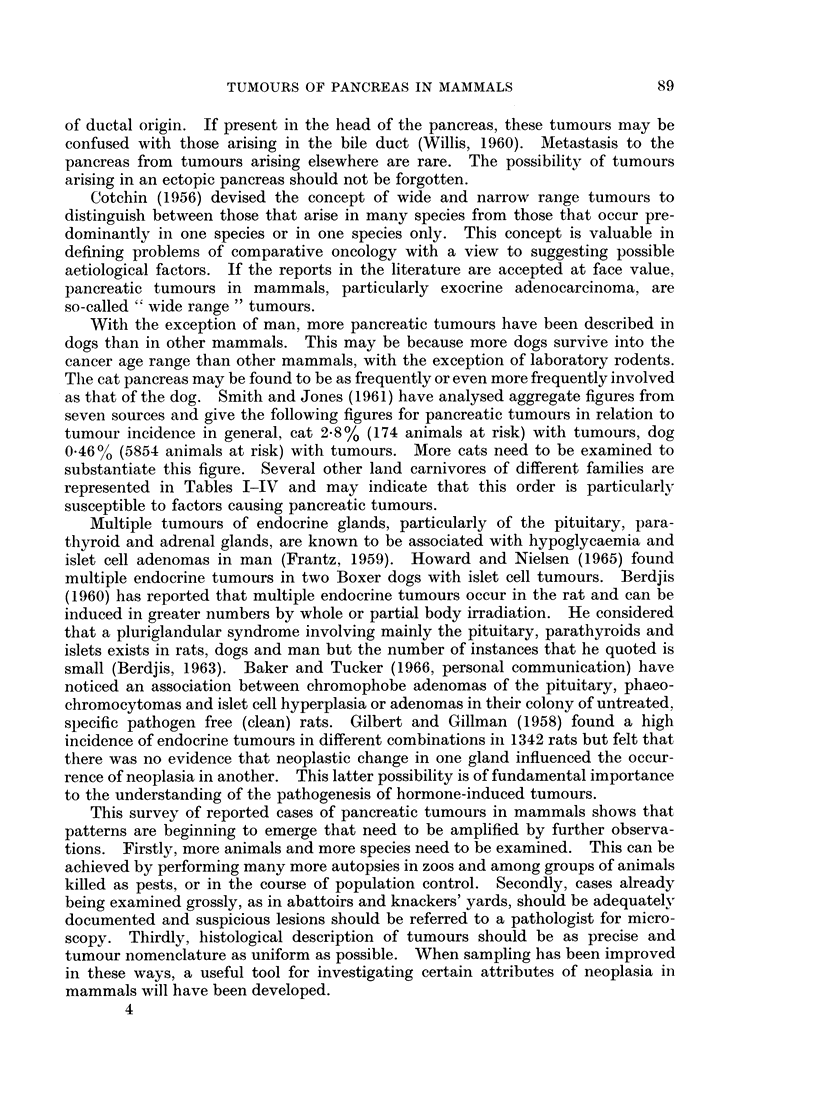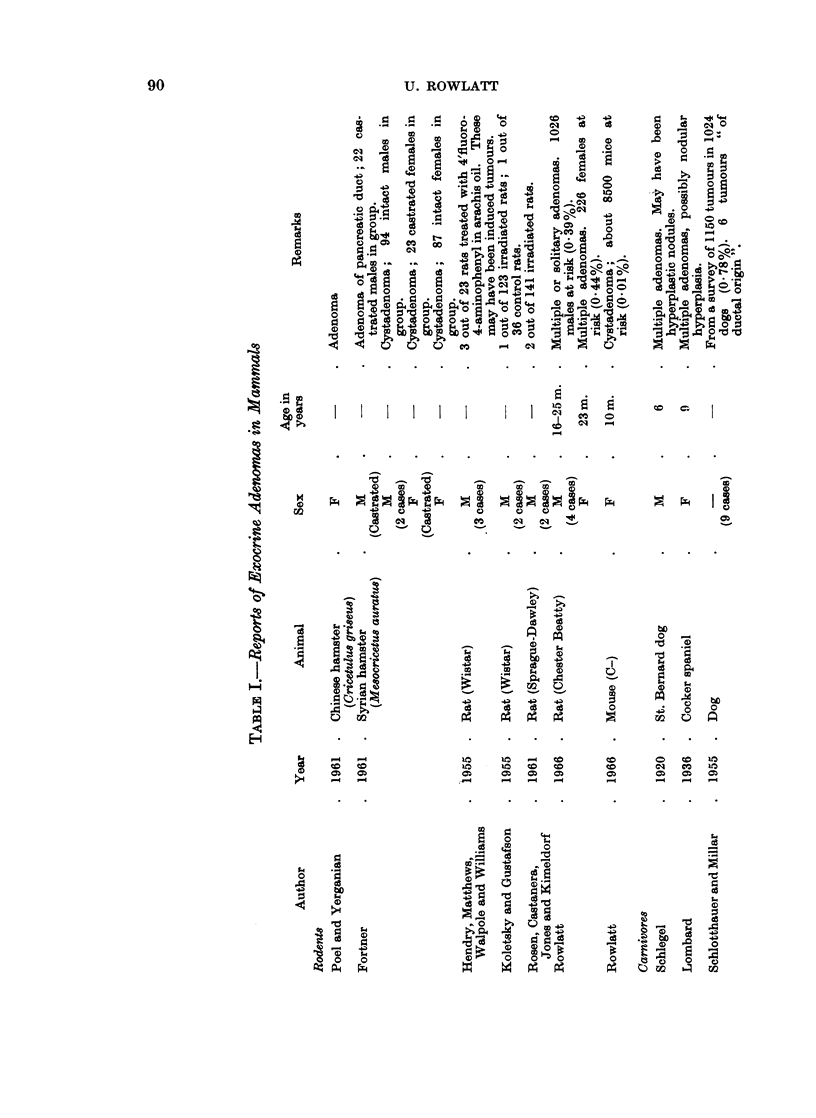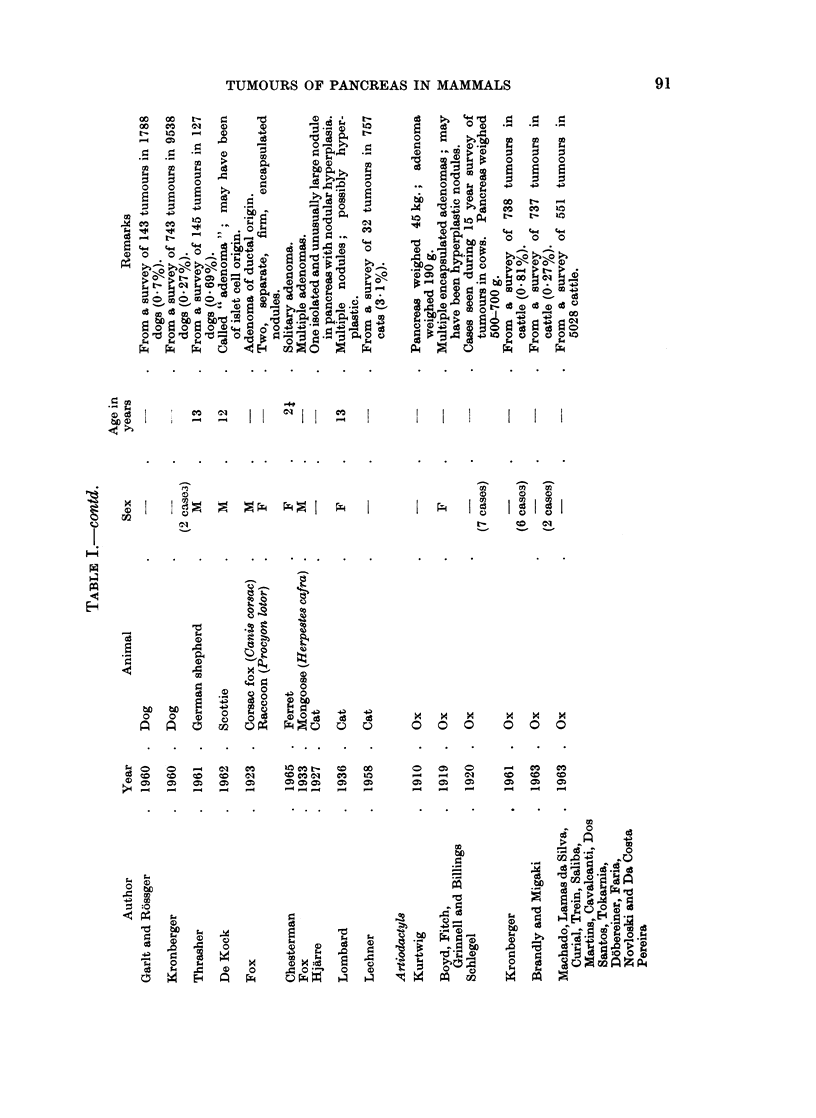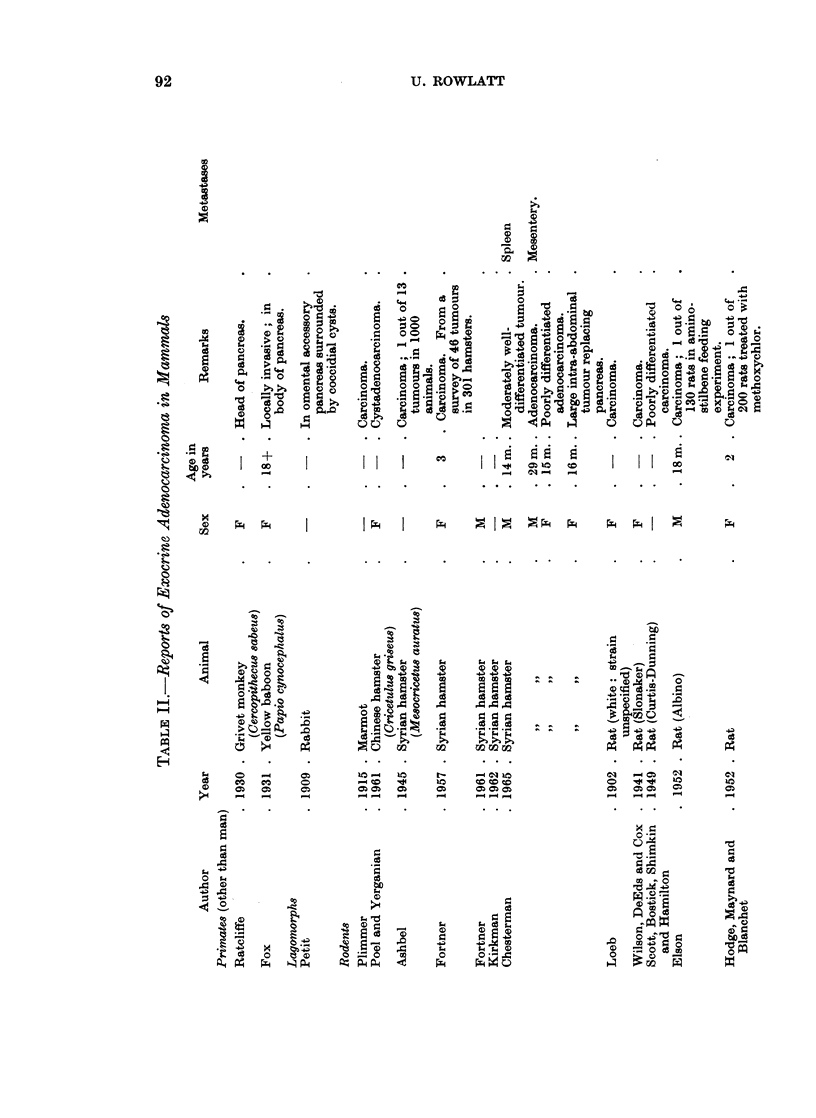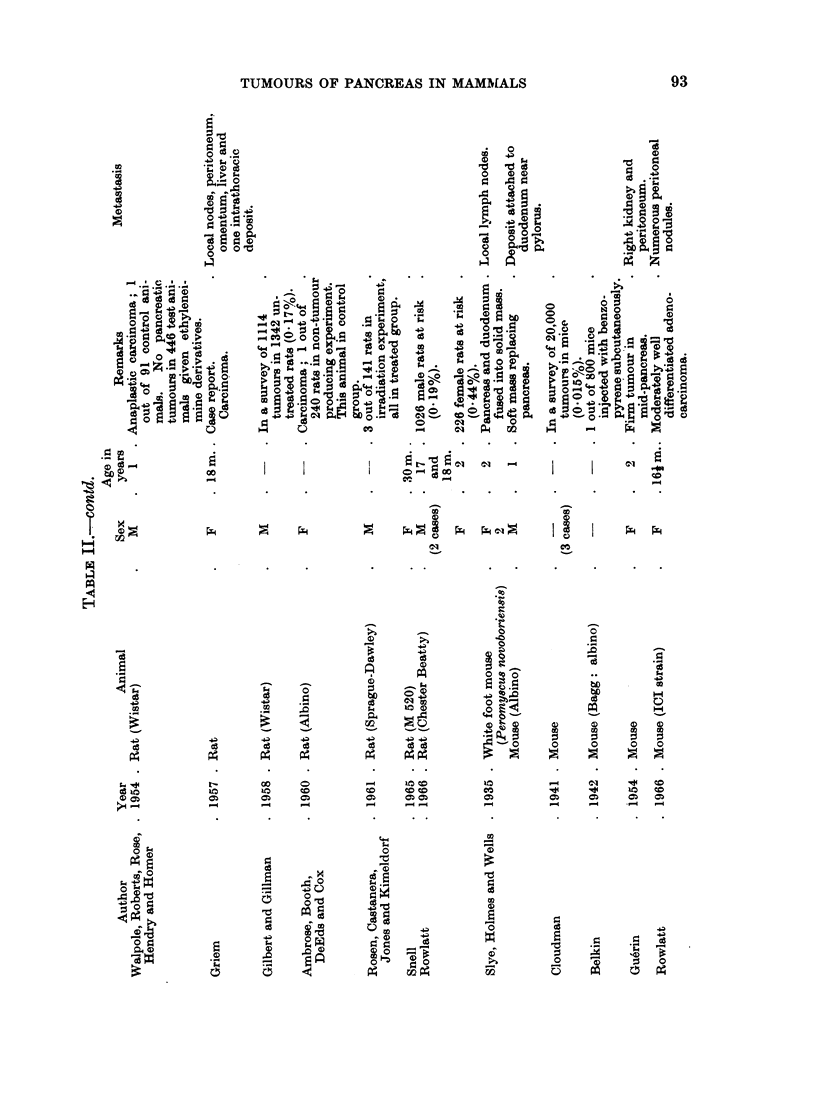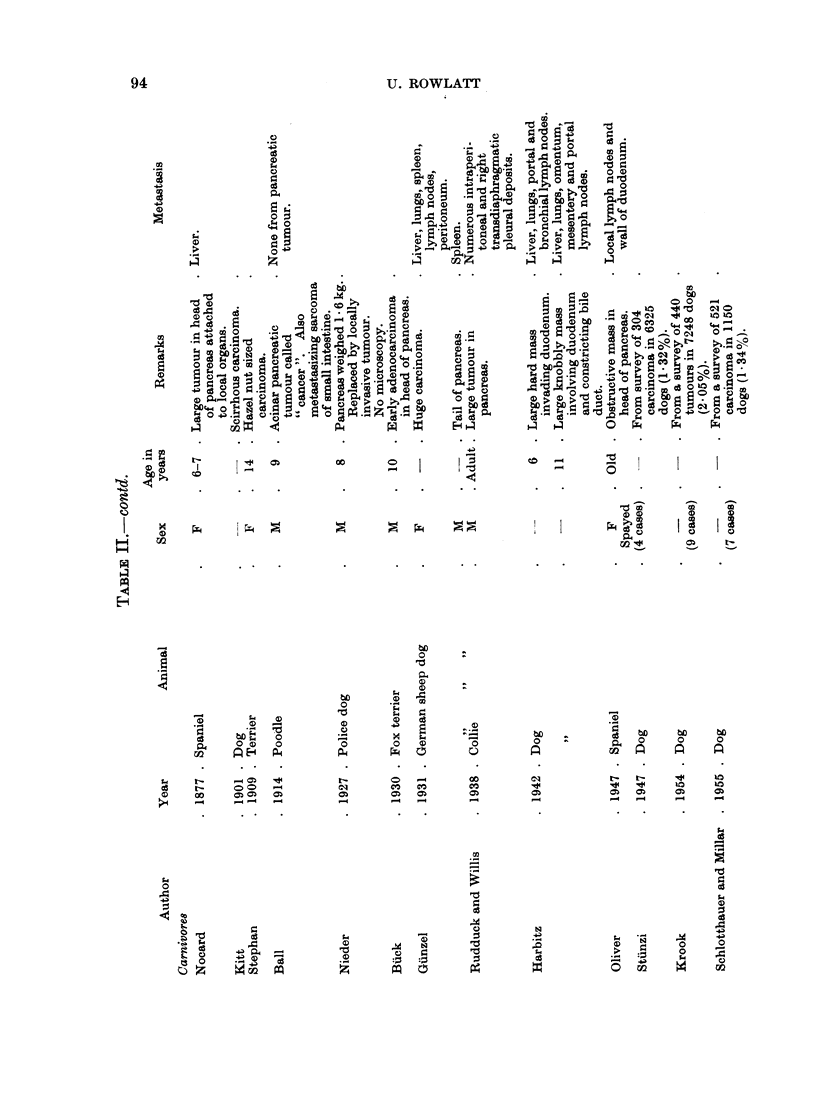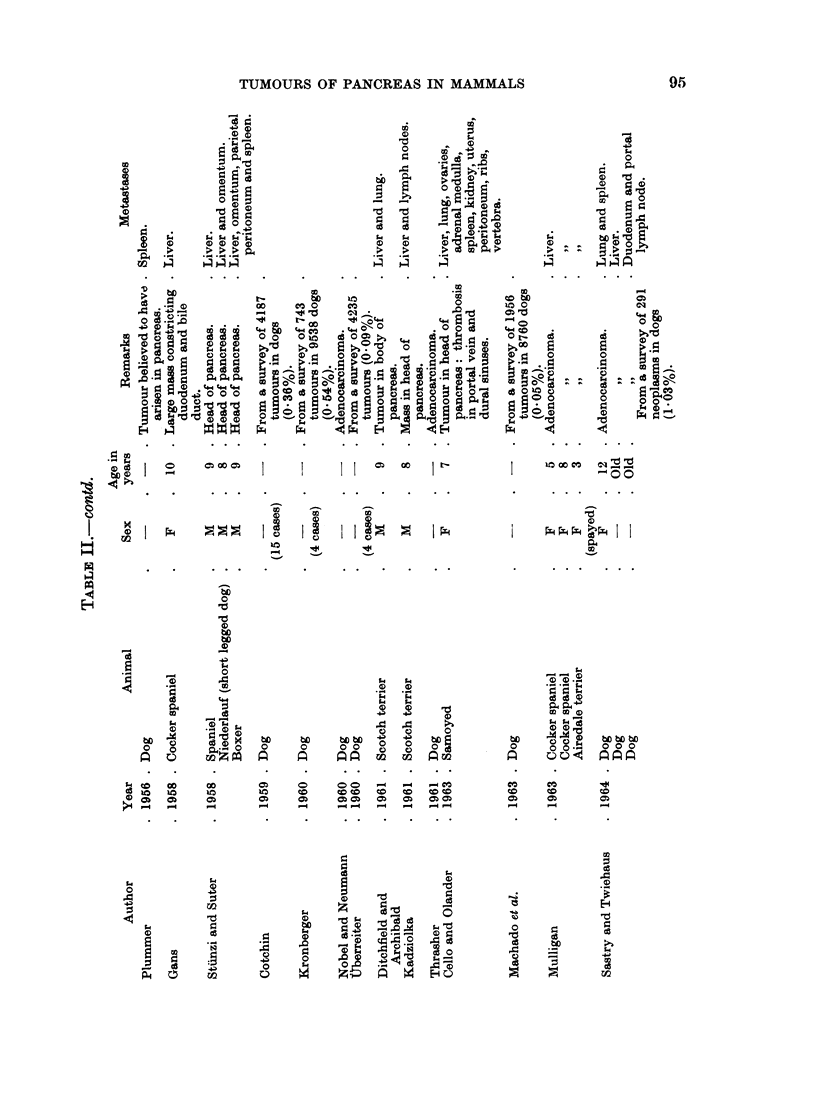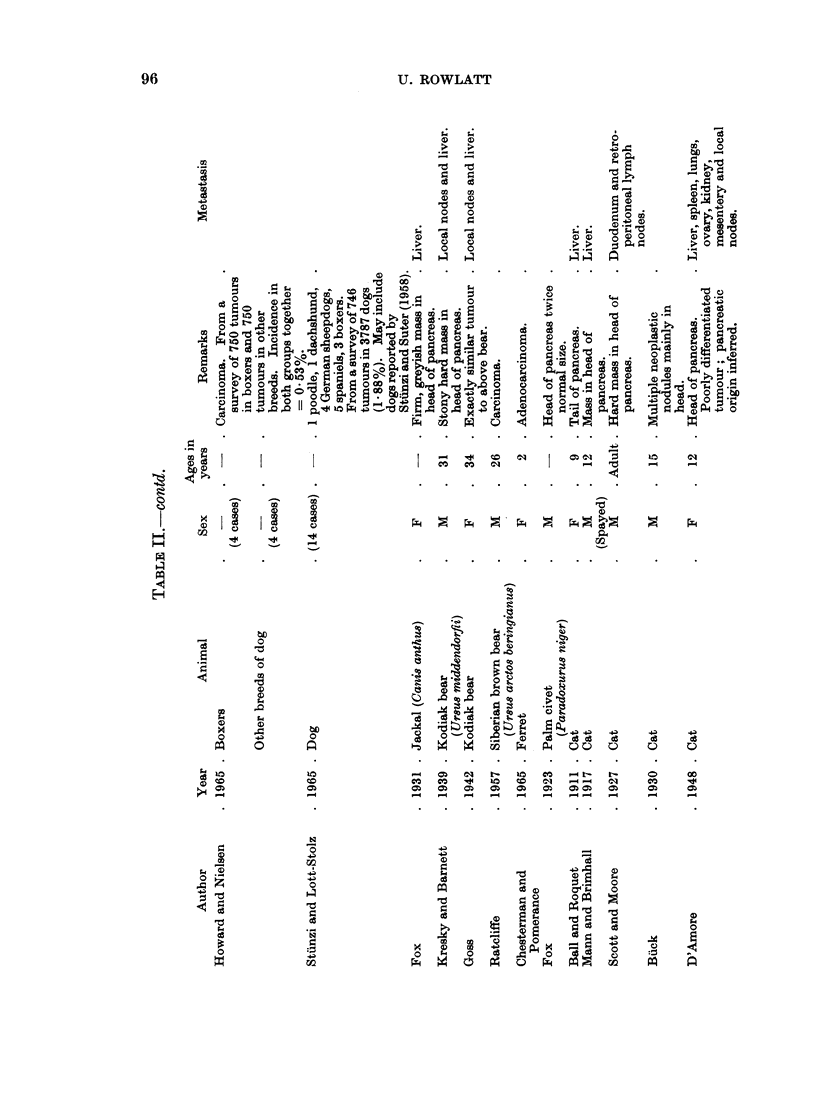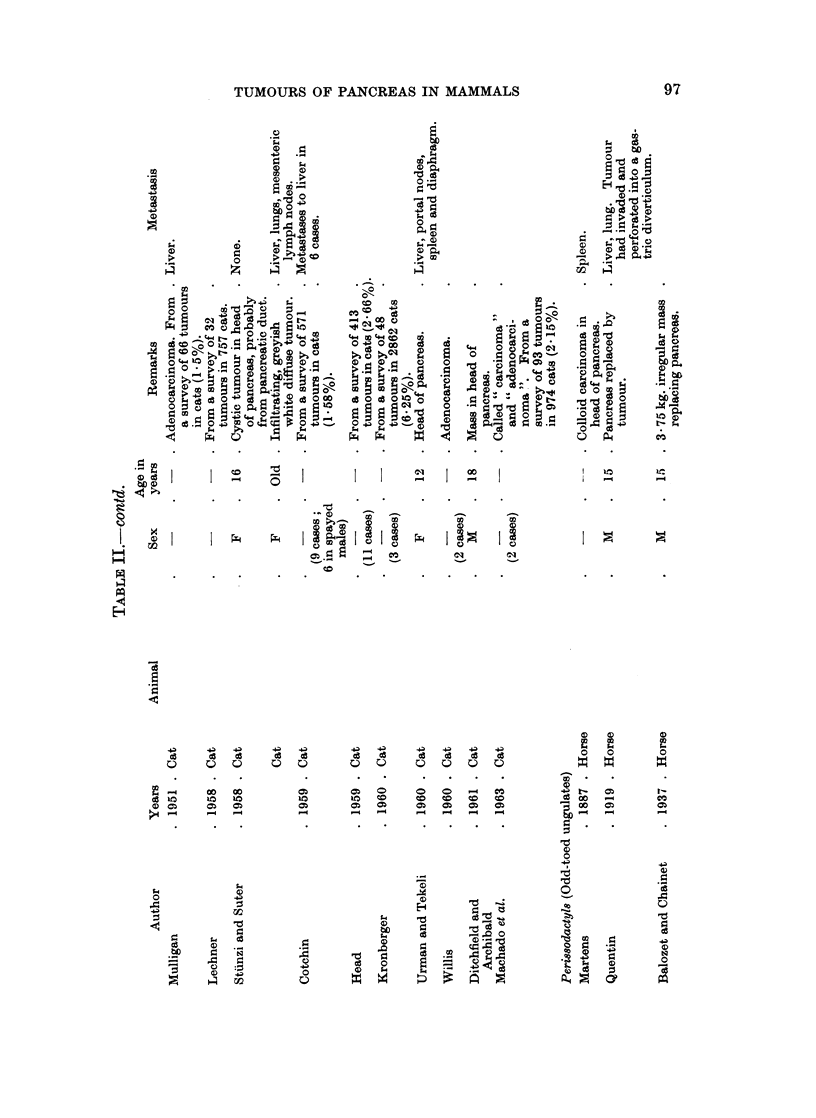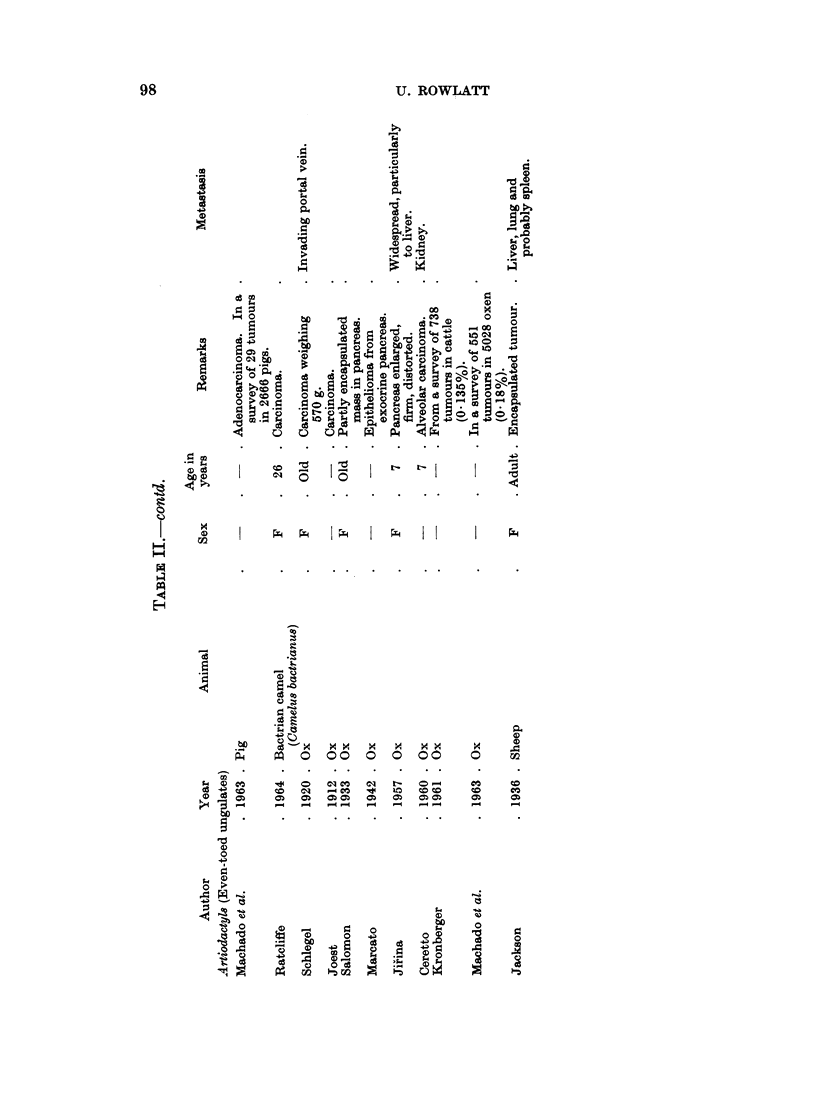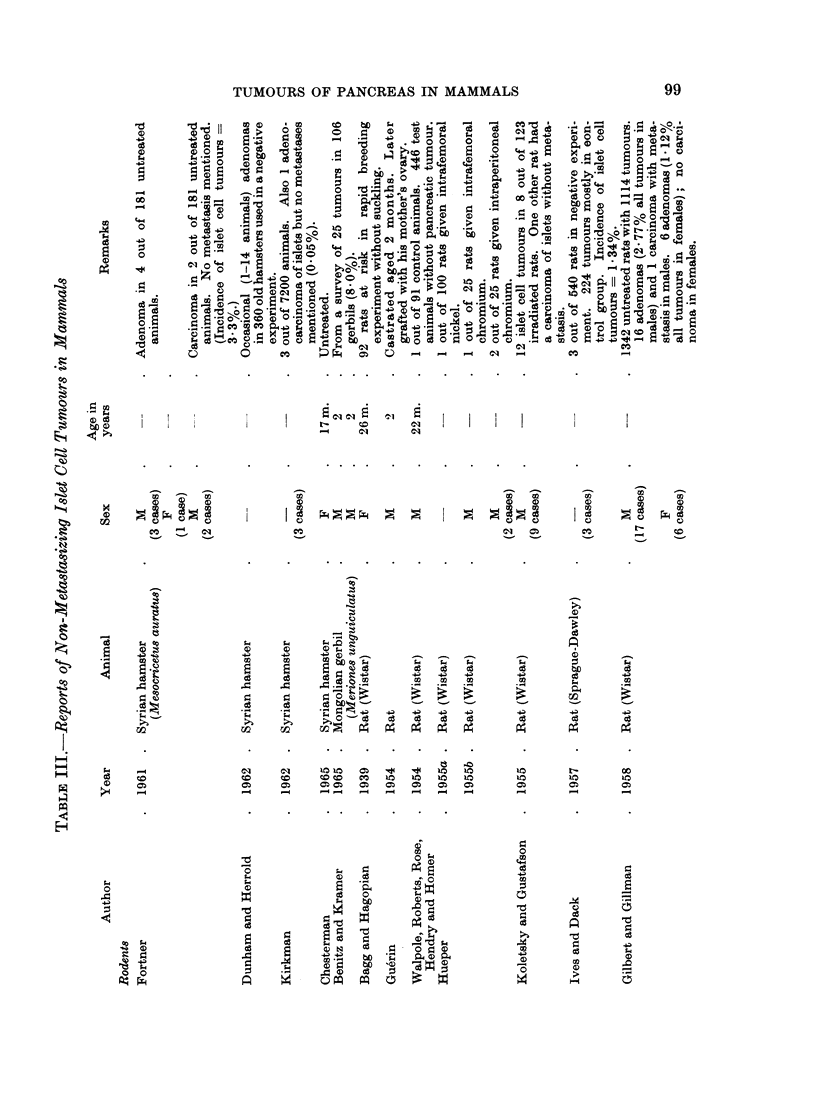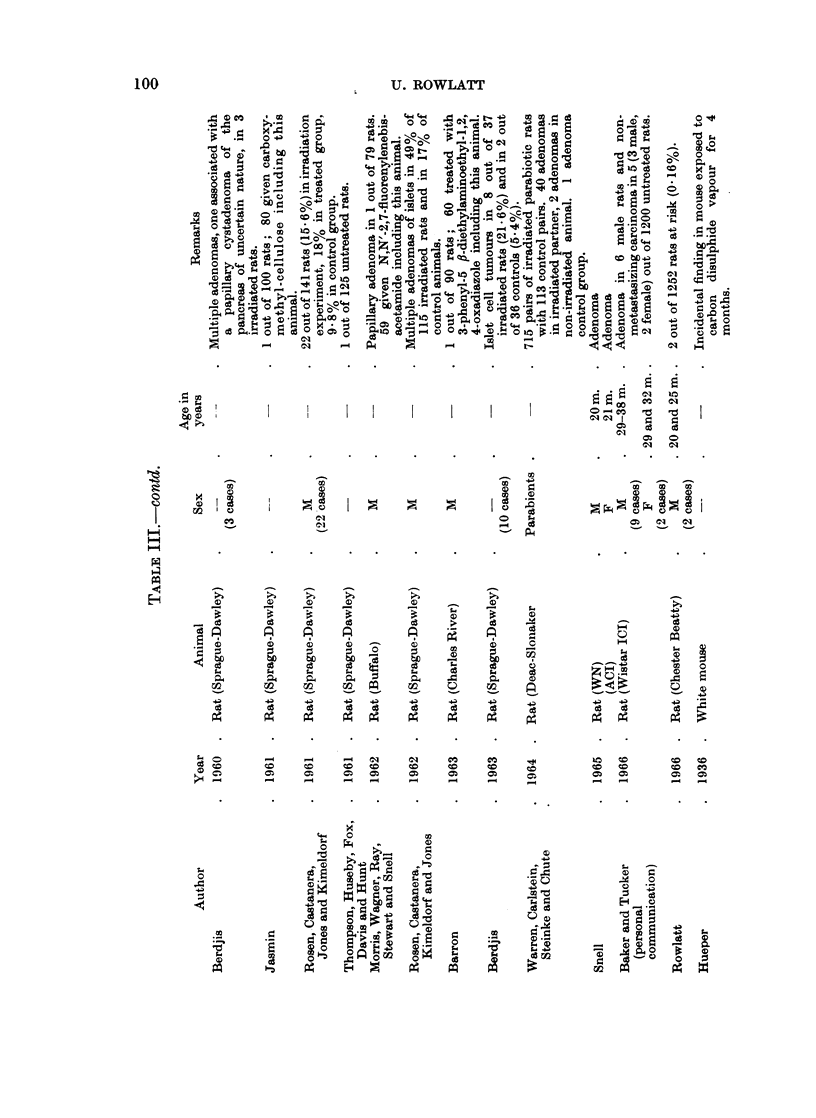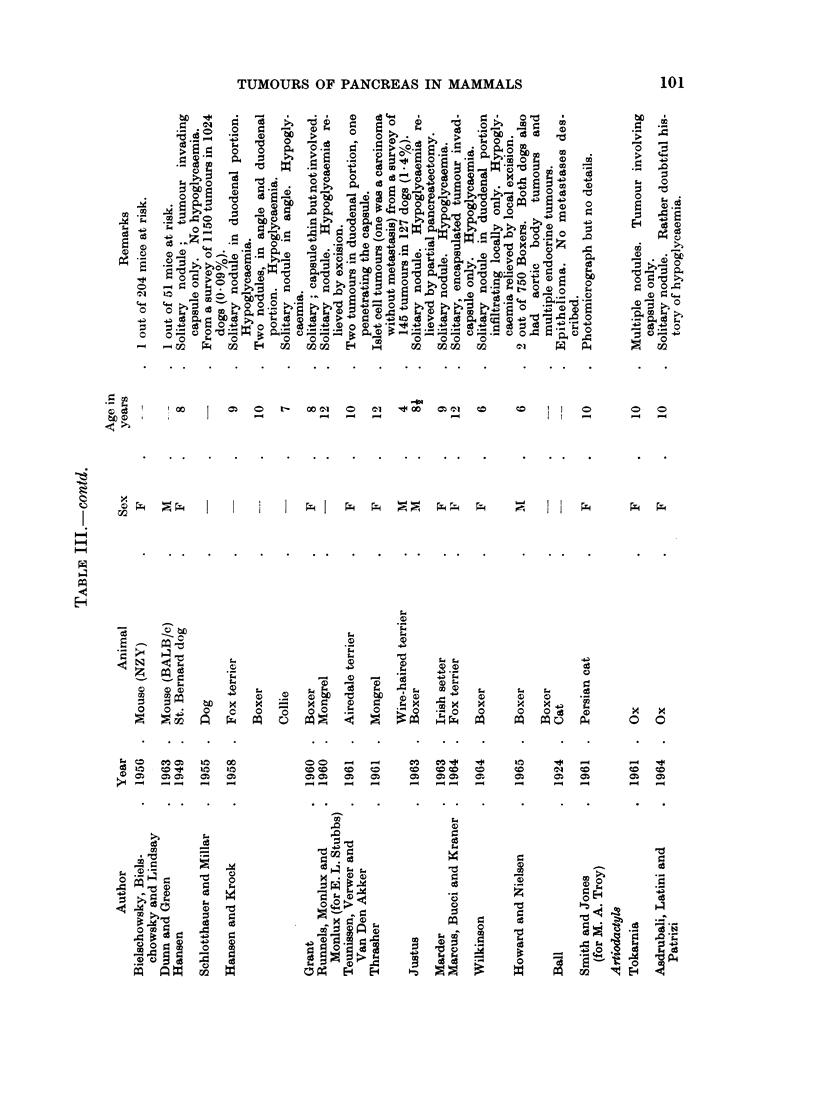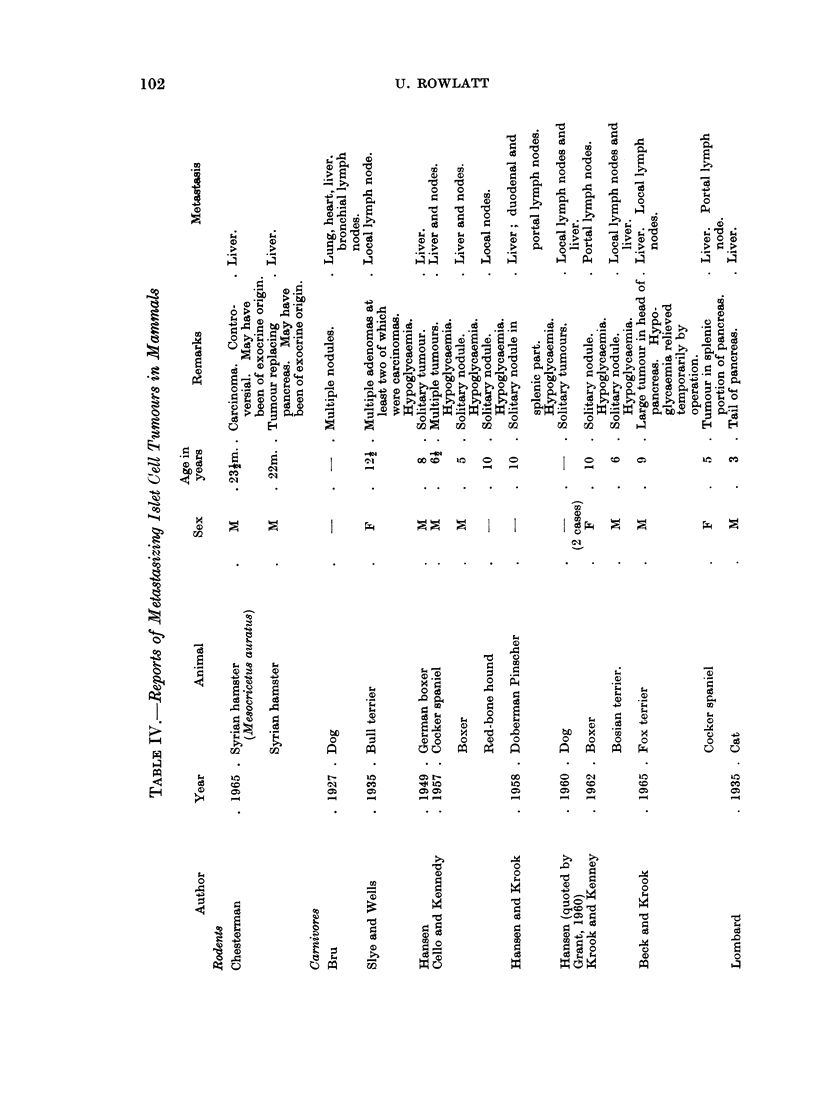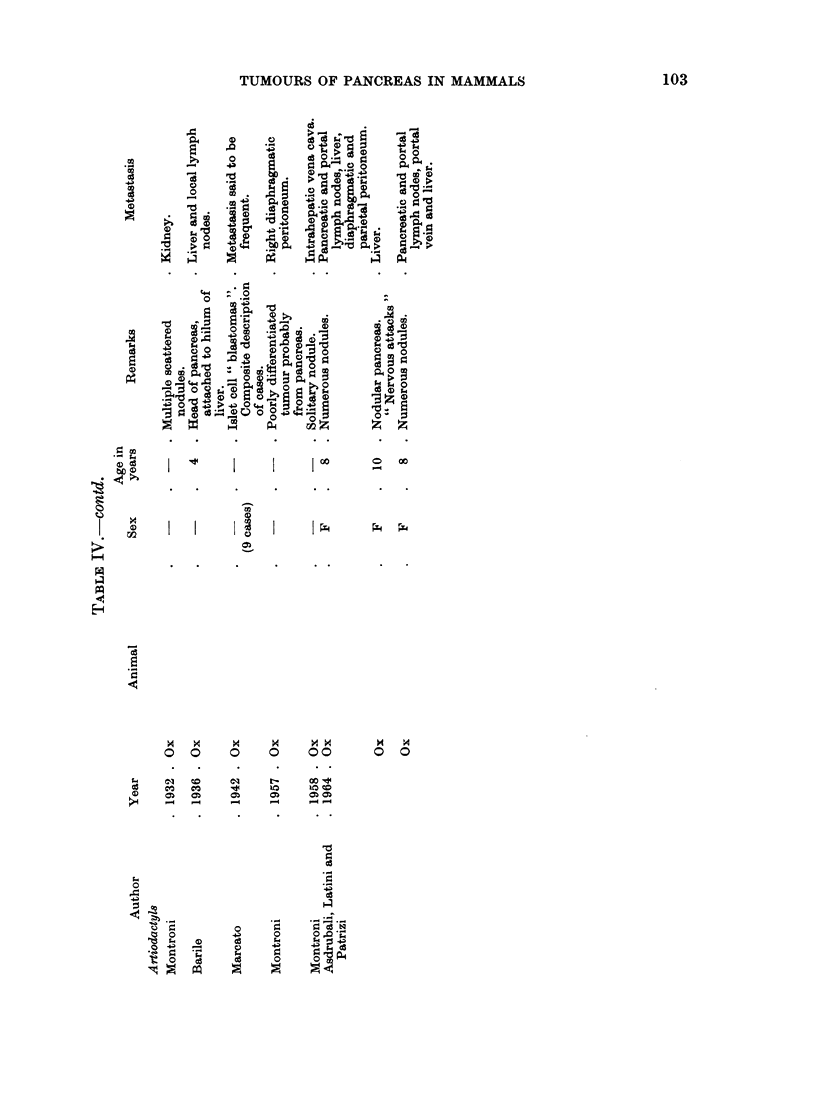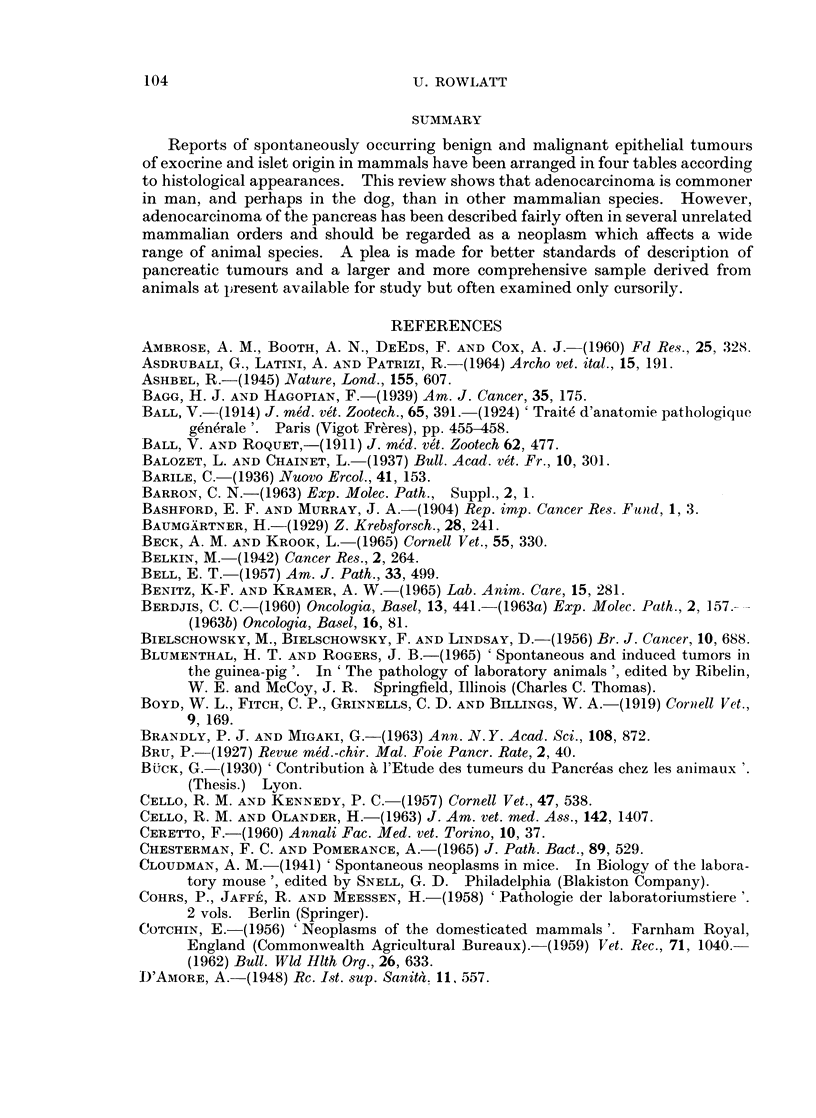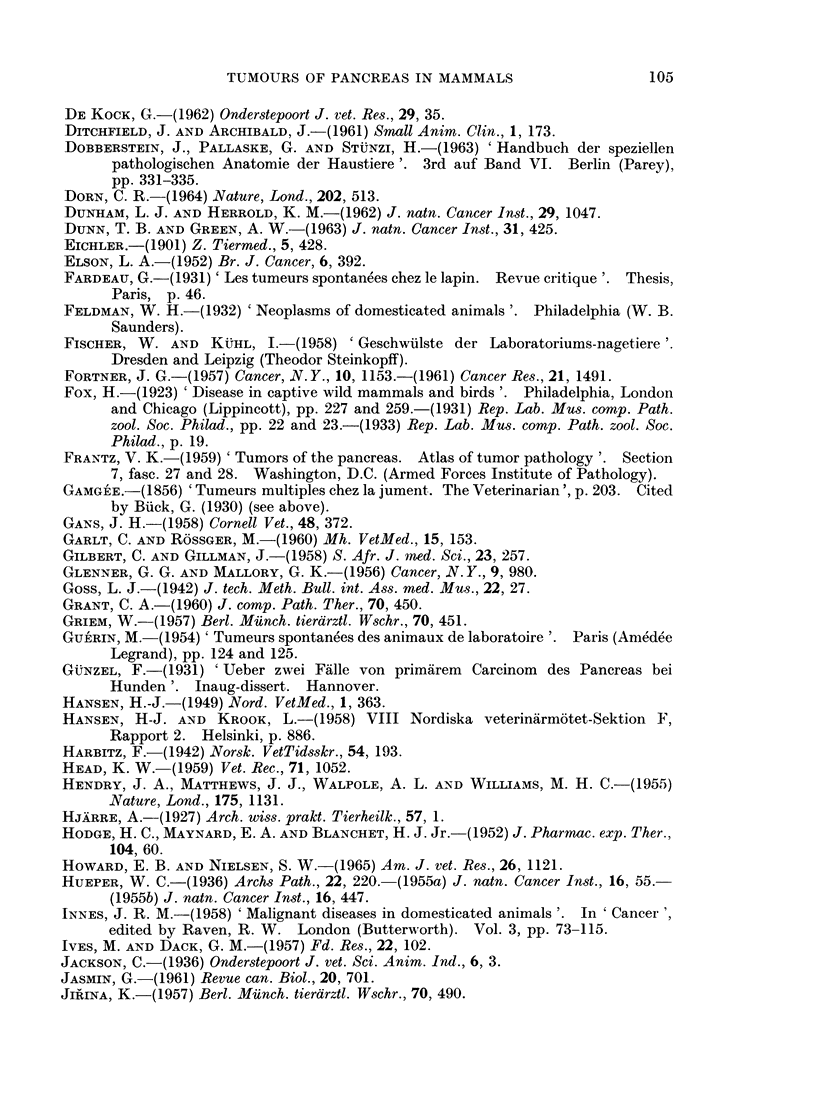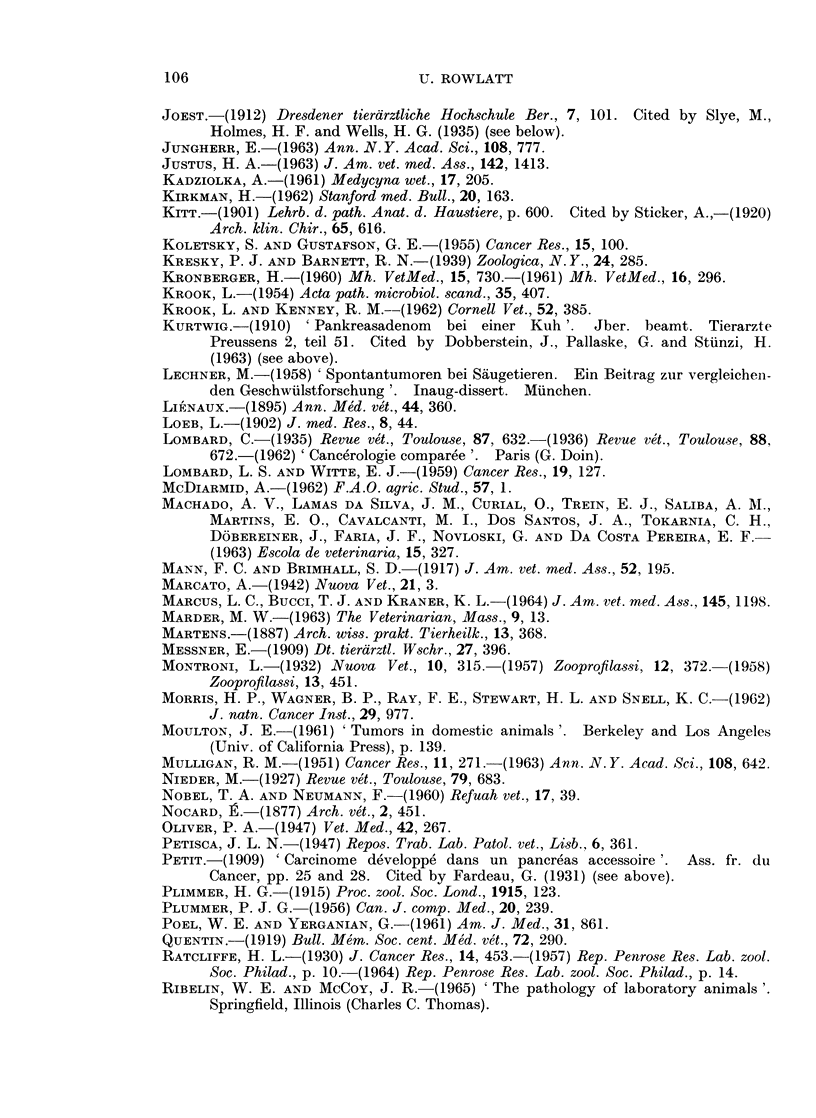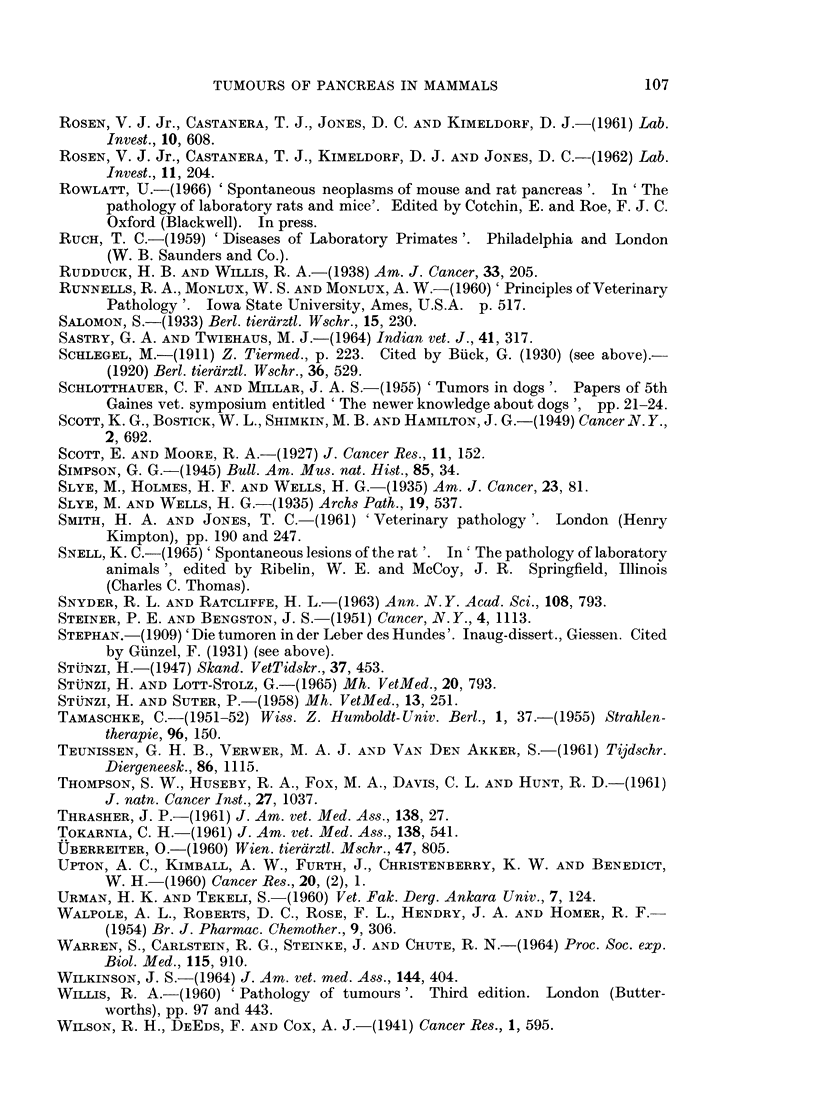# Spontaneous epithelial tumours of the pancreas of mammals.

**DOI:** 10.1038/bjc.1967.9

**Published:** 1967-03

**Authors:** U. Rowlatt


### SPONTANEOUS EPITHELIAL TUMOURS OF THE PANCREAS OF MAMMALS

U. ROWLATT

*From the Chester Beatty Research Institute, Institute of Cancer Research : Royal Cancer Hospital, Fulham Road, London, S.W.3*

Received for publication August 9, 1966

It has often been suggested that reviews of the reported incidence of spontaneous tumours in animals are of value in the search for aetiological agents responsible for cancer in man (Bashford and Murray, 1904 ; Feldman, 1932 ; Cotchin, 1956 ; Innes, 1958). Theories of causation of many human tumours should take into account the relative frequency of similar tumours in other mammals. The type of information collected together in these reviews is comparable with that given by epidemiological surveys and in the same way provides data for the study of patterns of incidence of neoplasia.

In addition to general reviews, a study of the comparative distribution of tumours of any particular organ may suggest carcinogenic mechanisms for that organ. Slye, Holmes and Wells (1935) collected together published reports of carcinoma of the pancreas in birds and mammals and found that the incidence was much higher in man than in other species. Tokarnia (1961) reviewed the reports of islet cell tumours in mammals and emphasized that cattle as well as dogs and man may develop hormone-secreting tumours. Many more reports of exocrine and endocrine tumours have accumulated since these papers were written and carcinoma of the pancreas should not be regarded as " very rare or not reported " as suggested by Innes (1958). The significance of these findings is still not understood but collection of accurate data should precede any attempt at interpretation.

Many difficulties arose in the compilation of the following tables based on reports of tumours in the literature. Some of the reports were fully documented and held conviction, others were recorded carefully but the available information was incomplete. One author used the services of six different pathologists in constructing what purported to be a homogeneous account of tumour incidence in his material (Thrasher, 1961). Some reports were no fuller than a bald record of " carcinoma of the pancreas " ; some were in the form of aggregate figures from large surveys of animal tumours arranged according to organ. One interesting finding was that certain tumours were described more frequently from some countries than others. Nearly all the reports of bovine islet cell tumours have come from Italy ; much of the early work on canine islet cell tumours was reported from Sweden. Composite reports such as those of Tamaschke (1951–52) and Smith and Jones (1961) have been omitted from the following tables in case the same animals were recorded twice.

Possibly the greatest drawback in any analysis based on reported data is that only a small percentage of the total number of animals available for autopsy are examined properly or at all. Cotchin (1962) has described the difficulties of collecting information about the nature and incidence of spontaneously occurring neoplasms in domesticated animals, the most important of which are non-reference of material to a pathologist and lack of proper background information about cases. The number of published post mortem studies on animals dying in zoos throughout the world is depressingly small and only a few are concerned particularly with tumours (for example Lombard and Witte, 1959, and Snyder and Ratcliffe, 1963, from the Philadelphia Zoological Gardens). McDiarmid (1962) has reviewed the sparse literature on tumours in free-living wild animals. The incidence of neoplasms in wild or feral British mammals destroyed as pests is said to be low (McDiarmid, 1966, personal communication) but figures are not available for study. Much useful information concerning spontaneous tumours in laboratory animals is buried in the extensive experimental pathology literature from which it can only be retrieved with difficulty. However, reviews are prepared from time to time and are invaluable for reference (Guérin, 1954 ; Tamaschke, 1955 ; Cohrs, Jaffé and Meessen, 1958 ; Fischer and Kühl, 1958 ; Ribelin and McCoy, 1965).

In spite of these obstacles, an attempt has been made to collect together all published records of spontaneous epithelial tumours of the pancreas in mammals. These reports have been listed in Tables I–IV according to histological type. The arrangement of the mammalian orders and their subdivisions follows that of Simpson (1945).

*Primates*

*Monkeys.*—Tumours in general are rare or rarely reported in monkeys ; a 0·08% incidence in 12,000 monkeys is given by Ruch (1959). Two pancreatic adenocarcinomas have been described in captive monkeys (Ratcliffe, 1930 ; Fox, 1931). No cases have been reported from the London Zoo and none are listed by Jungherr (1963). As yet no reports of exocrine or islet tumours have emerged from the increasing number of laboratory colonies of various species of monkey nor from the newly established primate centres in the United States.

*Man.*—The best review of pancreatic neoplasms in man is that of Frantz (1959). Benign tumours of exocrine origin are usually cystadenomas. Acinar adenomas are exceedingly rare if they occur at all (Glenner and Mallory, 1956). The commonest tumour is an adenocarcinoma of exocrine tissue. Bell (1957) reported an incidence of 1·23% in 33,367 males and 1·10% in 15,531 females over the age of 40 years. If only deaths from carcinoma were considered in this age group, the incidence was 5·5% for males and 4·3% for females. The head of the pancreas was involved in 59·1% of the cases, the body in 18·2%, the tail in 7·4% and the whole organ diffusely in 15·3%. Frantz was unwilling to give figures for islet cell tumours because of the ease with which they may be overlooked but quoted Korpássy's figure of 4 in 500 autopsies in which the pancreas was studied specially. Frantz emphasized the difficulty in assessing the malignancy of islet cell tumours because the histological evidence of invasiveness is not always correlated with the biological behaviour of the tumour.

*Lagomorphs*

*Rabbit.*—Only one rather doubtful case of pancreatic tumour has been reported in a rabbit in which a carcinoma surrounded by coccidial cysts was found in an accessory pancreas in the mesentery (Petit, 1909). This case was accepted as an adenocarcinoma by Fardeau (1931) who reviewed 73 cases of spontaneous tumours from the literature. No further pancreatic tumours have been reported since (Lombard, 1962).

*Rodents*

*Golden or Syrian hamsters.*—The overall incidence of spontaneous tumours in the golden hamster has varied greatly from one report to another. Ashbel (1945) found 13 spontaneous tumours in 1000 individuals. Kirkman (1962) reported an incidence of tumours of all sites of 11·3% in 7200 animals with a 0·03% incidence of exocrine adenocarcinoma and a 0·04% incidence of non-metastasizing tumours of islet origin. Dunham and Herrold (1962) noticed an occasional islet cell adenoma in old laboratory hamsters used in tests for carcinogenicity where negative results were obtained. Fortner (1957) reported 46 tumours in 301 animals (15·3%) but in 1961 increased this figure to 81·9% in 94 untreated males and 60·9% in 87 untreated females. In these 181 animals there were 3 benign exocrine tumours of ductal origin, 1 adenocarcinoma and 6 islet cell tumours. The incidence was lower in 22 males and 23 females that had been castrated when young but sexually mature.

We have been able to study 7 pancreatic tumours from the golden hamster by kind permission of Dr. F. C. Chesterman of the Imperial Cancer Research Fund. In 4 instances we were able to make out an exocrine origin and in 1 instance an islet pattern in the tumour or its metastases. In the remaining 2 cases the cells were of epithelial origin but bore no clear evidence of the parent tissue nor were special stains helpful. This state of affairs is quite unlike our experience with rats in which the distinctive morphology of the normal islet was present in a grotesque but recognizable form in the islet cell tumours.

*Chinese, grey or striped hamster.*—This animal has not been used as extensively in research as the golden hamster and reports of pancreatic tumours are correspondingly rare. Poel and Yerganian (1961) reported an adenoma of the exocrine pancreas in a diabetes-prone strain of hamsters. The authors pointed out that a statistical association exists between carcinoma of the pancreas and diabetes in man but did not correlate this observation with the degree of islet cell replacement by tumour. Also, they produced no evidence that the hamster tumour-bearers were themselves suffering from diabetes. Carcinoma of the pancreas was found in progeny of this same stock bred at Harwell in Dr. C. E. Ford's cytogenetic unit at one time but the incidence has fallen in recent years (Ford, 1966 ; personal communication).

*Rat.*—A preliminary review of pancreatic tumours in the laboratory rat has been published elsewhere (Rowlatt, 1966) and a full account of the lesions in 10 Chester Beatty strain rats is in preparation. We believe that exocrine adenomas are probably commoner than the small number of reports in the literature suggests. The same is probably true of islet cell adenomas as shown by the large number of these tumours in untreated animals examined by Rosen, Castanera, Kimeldorf and Jones (1962) who were particularly interested in the pancreas and gave it special scrutiny.

Although some of the animals listed in the tables were used in experiments, the various agents to which they were exposed were not thought to have induced the tumours. The exception to this is the work of Hendry, Matthews, Walpole and Williams (1955) who took the view that a high yield of tumours in several organs including the pancreas in 23 rats was related to subcutaneous injection of 4′-fluoro-4-aminodiphenyl in arachis oil. The irradiation experiments of Koletsky and Gustafson (1955), Berdjis (1960 and 1963), Rosen, Castanera, Jones and Kimeldorf (1961) and the same team in 1962 and Warren, Carlstein, Steinke and Chute (1964) may have induced islet cell tumours.

*Mouse.*—All types of pancreatic tumours are rarer in mice than rats. As in the rat, the islet cell adenoma described by Upton, Kimball, Furth, Christenberry and Benedict (1960) may have been induced by irradiation.

*Guinea-pig.*—Reports of 140 spontaneously occurring tumours in guinea-pigs have been reviewed by Blumenthal and Rogers (1965). None were in the pancreas.

*Other rodents.*—A pancreatic adenocarcinoma in a marmot (woodchuck) was reported without histological details from the London Zoo (Plimmer, 1915) and is the only tumour of this organ in the Zoo's records. A review of 25 tumours in 106 Mongolian gerbils, a rodent species relatively new to research, showed an 8·0% incidence of non-metastasizing islet cell tumours (Benitz and Kramer, 1965).

### *Carnivores*

*Dog.*—Hyperplastic nodules without increase in size of the pancreas as a whole have been described in dogs (Moulton, 1961). Baumgärtner (1929) found multiple nodules varying from the size of a pinhead to that of a pea in 85% of dogs between 7 and 20 years of age. He regarded these nodules as a normal ageing change without clinical significance.

True adenomas have been reported several times, usually without histological description, with the exception of 9 tumours reported by Schlotthauer and Millar (1955), 6 of which were said to have been of ductal origin. The tumour said by De Kock (1962) to have stained paler than the surrounding pancreas may have been of islet cell origin.

The commonest tumour of the dog pancreas is an exocrine adenocarcinoma. If all neoplasms are included, the incidence of pancreatic carcinoma has been given as 0·36% (Cotchin, 1959), 0·54% (Kronberger, 1960) or 0·53% (Howard and Nielsen, 1965). Uberreiter (1960) and Machado and his colleagues (1963) give the lower figures of 0·09% and 0·05% respectively but Stünzi and Lott-Stolz (1965) report an incidence of 1·88% of all tumours in their material. If only carcinomas are considered, the figure is 1·32% (Stünzi, 1947), 1·34% (Schlotthauer and Millar, 1955) or 2·05% (Krook, 1954).

The tumour is usually scirrhous and arises more commonly in the midportion (or head) than in either the duodenal or splenic (tail) branches. Because of the anatomical association of the head of the pancreas with the terminal part of the bile duct, obstructive jaundice and biliary cirrhosis may complicate the usual clinical picture of pain, wasting and a palpable mass in the abdomen. Similarly, the portal vein or its branches (or even the posterior vena cava as in Nocard's case) may be compressed, with resulting ascites. Ascites may also be caused by multiple peritoneal seedling deposits as in the dog described by Rudduck and Willis (1938). Occasionally, the animal may present with signs related to a distant metastasis as in the dog with spinal cord compression described by Cello and Olander (1963). In discussing the pathogenesis of pancreatic adenocarcinoma,

Stünzi and Suter (1958) stated that it is usually agreed that such tumours are derived from the duct epithelium but they gave no corroborating details.

Islet cell tumours are rarer than those of the exocrine pancreas but are of greater importance because of the constitutional effects, particularly on the central nervous system, if the tumour is insulin-secreting (Krook and Kenney, 1962). The tumour may be solitary or multiple and arises more frequently in the duodenal branch than elsewhere in the pancreas (Grant, 1960). Hypoglycaemic attacks present as periodic epileptiform convulsions, weakness, ataxia and fainting associated usually with fasting or exertion (Cello and Kennedy, 1957 ; Hansen and Krook, 1958). Blood sugar levels of 30–40 mg.% have been reported during an attack. Such attacks are relieved temporarily by glucose or permanently by surgery (Runnels, Monlux and Monlux, 1960 ; Justus, 1963 ; Wilkinson, 1964) if operation is performed before metastasis has occurred.

*Cat.*—Reactive hyperplastic nodules have been described in cats by Hjärre (1927) and Lombard (1936). Many fewer cases of true benign or malignant tumours of exocrine or endocrine origin have been reported in cats than dogs but fewer animals have been examined. Adenocarcinoma arises more often in the head than in the tail of the pancreas. Biliary cirrhosis has been reported by Ball and Roquet (1911), Mann and Brimall (1917) and Stünzi and Suter (1958), and ascites due to extensive lymphatic obstruction by Scott and Moore (1927). Metastasis to the liver is common (Cotchin, 1959). There appear to have been no reports of hypoglycaemia due to functioning islet cell tumours in cats.

*Other carnivores.* It is curious that pancreatic tumours have been described in six out of the seven families of carnivores, the exception being the hyaenas, from which group only a few individuals have been examined. An adenoma in a fox and an adenocarcinoma in a jackal have been described in wild canids (Fox, 1923 and 1931). The viverrids are represented by a mongoose with multiple exocrine adenomas (Fox, 1933) and a palm civet with a carcinoma of the head of the pancreas (Fox, 1923). Two exocrine adenomas in a raccoon are the only tumours reported in procyonid carnivores (Fox, 1923). Two cases of exocrine tumour have been described in mustelids (both in ferrets), one being an adenocarcinoma (Chesterman and Pomerance, 1965) and the other an adenoma found incidentally in an enlarged but otherwise normal pancreas (Chesterman, 1965, personal communication). Three exocrine adenocarcinomas have been described in bears, one in a Siberian bear (Ratcliffe, 1957) and two in Kodiak bears (Kresky and Barnett, 1939 ; Goss, 1942). The last pair of animals were caught on Kodiak Island as cubs and shared the same cage in the New York Zoo for 31 years. Both died from their pancreatic tumours and were found to have identical metastases. Dorn (1964) has reported carcinoma of the liver in 5 out of 6 bears who shared the same enclosure in the San Diego Zoo for long periods of their lives.

### *Perissodactyls*

*Horse.*—The multiple tumours found by Gamgée (1856) in the pancreas of an aged mare may have been hyperplastic nodules. Adenocarcinoma has been reported three times in the horse. Martens (1887) described a colloid carcinoma in the head of the pancreas with obstruction of the common bile duct. This was the only pancreatic tumour in 213 tumours of horses reviewed by Eichler (1901). The pancreas was totally replaced by tumour in the cases reported by Quentin

(1919) and Balozet and Chainet (1937) ; in both instances a pancreatic origin was inferred. Kronberger (1961) reviewed 1016 tumours from horses, from material examined at the Institute of Veterinary Pathology in Leipzig between 1917 and 1959. He found one sarcoma and one melanosarcoma but no epithelial tumours of the pancreas in this material.

*Artiodactyls*

*Oxen.*—Multiple adenomas have been described several times in cattle but the distinction between nodular hyperplasia and true neoplastic tumours had not always been made clear (Liénaux, 1895 ; Messner, 1909 ; Schlegel, 1911 ; Boyd, Fitch, Grinnells and Billings, 1919 ; Jackson, 1936). The bovine nodular pancreas, unlike that of the dog, may be greatly enlarged as in the animal reported by Schlegel (1911) in which the pancreas was three times the normal size. The same author reported a weight range for the pancreas of 500–700 g. in 7 cows seen during a 15 year survey of tumours in cows (Schlegel, 1920). Probably the tumour weighing 190 g. in a pancreas weighing 45 kg. reported by Kurtwig (1910) may have been alarge hyperplastic nodule. The adenoma described by Petisca (1947) was said to be in a ruminant, which may have been an ox.

Many surveys of tumours in domesticated and farm animals have been considered in the preparation of the present review but only 8 reports of adenocarcinoma in cattle have been found. Steiner and Bengston (1951), among others, emphasized that food-producing animals are usually killed before reaching an age when carcinomas may be expected. Many calves, cattle and steers are under, near or slightly over the age of puberty and in good health at the time of slaughter.

Several cases of metastasizing and non-metastasizing tumours of the islets in cattle have been reported from Italy. The histological reports carry conviction ; confusion with non-neoplastic changes seems unlikely. Some of the tumours were thought to be hypersecretory, based on non-specific nervous symptoms but in the absence of blood sugar readings, the matter remains unproved. Tokarnia (1961) reported spasmodic attacks of limb stiffening, eye rolling and head retraction in a cow which was subsequently found to have a non-metastasizing islet cell tumour. The animal died before a blood sugar level could be measured but the author in reviewing accounts of experimentally produced hypoglycaemia in cattle considered that the convulsive signs were compatible with hyperinsulinism.

*Other artiodactyls.*—No pancreatic tumours have been reported in goats or pigs and only one adenocarcinoma in a sheep (Jackson, 1936). An adenocarcinoma in a camel was described by Ratcliffe (1964).

### DISCUSSION

The most pressing need in the study of spontaneous tumours of the pancreas is for precision of diagnosis or, if such is impossible, for accurate verbal and photographic record of pathological specimens. Several questions need to be answered :

(1) if *benign*, is the tumour a true adenoma or a compensatory hyperplastic nodule? Is it of exocrine or islet origin? If exocrine, did it arise from ducts or acini or both?

(2) if *malignant*, what is the degree of cellular pleomorphism and the extent of invasion? What are the grounds for regarding the tumour as of pancreatic origin? Can the possibility of a bile duct tumour be eliminated? Is the pancreas considered to be the site of origin of the tumour only because other organs are not involved but the pancreas totally replaced by tumour?

As in the case of liver tumours, it is difficult to distinguish between hyperplastic nodules and benign autonomous neoplasms. Features such as a large size, a small number of lesions, the presence of a capsule and the absence of atrophy or post-inflammatory changes elsewhere in the pancreas suggest that the lesion is an adenoma rather than a reactive hyperplastic nodule. Some animals such as aged dogs, cats and cattle seem to form compensatory pancreatic nodules more frequently than others.

Islet cell adenomas may be distinguished from exocrine tumours by architectural construction and by staining properties. Pale-staining cells arranged on a rich sinusoidal network, hydropic degeneration of cytoplasm, and many small haemorrhages within the tumour with consequent deposition of iron pigment in the stroma are characteristic of islet cell tumours. These tumours retain many of the features of a normal islet. There appears to be no method by which a small adenoma may be distinguished from a giant islet with certainty.

Special stains may be used to classify pancreatic adenomas. Zymogen granules present in exocrine adenomas stain with the trichrome methods of Masson or Mallory. Gomori's aldehyde fuchsin method has been widely recommended for staining islet $\beta$ cell granules but this stain needs to be freshly prepared and is less efficient for some animal material than for others. Chrome-haematoxylin phloxine is said to give uneven results (Grant, 1960). Mucin stains should identify cells arising from the main pancreatic duct but a normal control piece cf duct from the animal under investigation should be included. In our experience, special stains are of greater use in confirming than establishing a diagnosis.

Although it is true that all epithelial components of the pancreas arise from the same endodermal bud, and that acinar and islet cells can be formed post-natally from duct cells in response to various stimuli (Frantz, 1959), typical tumours arising from each element are recognizable. Undoubtedly, controversial tumours that cannot be classified with certainty will occur but these are probably relatively uncommon as are tumours that are undoubtedly mixed. Hyperplasia of islet tissue may accompany adenocarcinoma of exocrine glands : this may explain why diabetes mellitus is an unusual symptom although there may be widespread destruction of the pancreas (Stünzi and Suter, 1958).

Criteria of malignancy should be clearly stated. The presence of even a small patch of cellular atypism and an increase in mitotic figures in an adenoma may be taken to indicate malignancy by some pathologists. Others require invasion of the adjacent normal pancreas. Metastases, when present, confirm the diagnosis but the animal may have been killed for other reasons before distant spread had occurred. Criteria of malignancy in the case of islet cell tumours also vary from pathologist to pathologist. We follow Frantz (1959) in regarding distant spread as necessary proof of malignancy because of the non-correlation between traditional histological evidence of local invasion and subsequent behaviour of the tumour. In view of this, we have listed all islet cell tumours from the literature as metastasizing or non-metastasizing tumours.

Most adenocarcinomas of the domestic carnivores and of man are thought to be of ductal origin. If present in the head of the pancreas, these tumours may be confused with those arising in the bile duct (Willis, 1960). Metastasis to the pancreas from tumours arising elsewhere are rare. The possibility of tumours arising in an ectopic pancreas should not be forgotten.

Cotchin (1956) devised the concept of wide and narrow range tumours to distinguish between those that arise in many species from those that occur predominantly in one species or in one species only. This concept is valuable in defining problems of comparative oncology with a view to suggesting possible aetiological factors. If the reports in the literature are accepted at face value, pancreatic tumours in mammals, particularly exocrine adenocarcinoma, are so-called " wide range " tumours.

With the exception of man, more pancreatic tumours have been described in dogs than in other mammals. This may be because more dogs survive into the cancer age range than other mammals, with the exception of laboratory rodents. The cat pancreas may be found to be as frequently or even more frequently involved as that of the dog. Smith and Jones (1961) have analysed aggregate figures from seven sources and give the following figures for pancreatic tumours in relation to tumour incidence in general, cat 2·8% (174 animals at risk) with tumours, dog 0·46% (5854 animals at risk) with tumours. More cats need to be examined to substantiate this figure. Several other land carnivores of different families are represented in Tables I–IV and may indicate that this order is particularly susceptible to factors causing pancreatic tumours.

Multiple tumours of endocrine glands, particularly of the pituitary, parathyroid and adrenal glands, are known to be associated with hypoglycaemia and islet cell adenomas in man (Frantz, 1959). Howard and Nielsen (1965) found multiple endocrine tumours in two Boxer dogs with islet cell tumours. Berdjis (1960) has reported that multiple endocrine tumours occur in the rat and can be induced in greater numbers by whole or partial body irradiation. He considered that a pluriglandular syndrome involving mainly the pituitary, parathyroids and islets exists in rats, dogs and man but the number of instances that he quoted is small (Berdjis, 1963). Baker and Tucker (1966, personal communication) have noticed an association between chromophobe adenomas of the pituitary, phaeochromocytomas and islet cell hyperplasia or adenomas in their colony of untreated, specific pathogen free (clean) rats. Gilbert and Gillman (1958) found a high incidence of endocrine tumours in different combinations in 1342 rats but felt that there was no evidence that neoplastic change in one gland influenced the occurrence of neoplasia in another. This latter possibility is of fundamental importance to the understanding of the pathogenesis of hormone-induced tumours.

This survey of reported cases of pancreatic tumours in mammals shows that patterns are beginning to emerge that need to be amplified by further observations. Firstly, more animals and more species need to be examined. This can be achieved by performing many more autopsies in zoos and among groups of animals killed as pests, or in the course of population control. Secondly, cases already being examined grossly, as in abattoirs and knackers' yards, should be adequately documented and suspicious lesions should be referred to a pathologist for microscopy. Thirdly, histological description of tumours should be as precise and tumour nomenclature as uniform as possible. When sampling has been improved in these ways, a useful tool for investigating certain attributes of neoplasia in mammals will have been developed.

TABLE I.—*Reports of Exocrine Adenomas in Mammals*

| Author | Year | Animal | Sex | Age in years | Remarks |
|---|---|---|---|---|---|
| *Rodents* | | | | | |
| Poel and Yerganian | 1961 | Chinese hamster (*Cricetulus griseus*) | F | — | Adenoma |
| Fortner | 1961 | Syrian hamster (*Mesocricetus auratus*) | M (Castrated) | — | Adenoma of pancreatic duct ; 22 castrated males in group. |
| | | | M (2 cases) | — | Cystadenoma ; 94 intact males in group. |
| | | | F (Castrated) | — | Cystadenoma ; 23 castrated females in group. |
| | | | F | — | Cystadenoma ; 87 intact females in group. |
| Hendry, Matthews, Walpole and Williams | 1955 | Rat (Wistar) | M (3 cases) | — | 3 out of 23 rats treated with 4′fluoro-4-aminophenyl in arachis oil. These may have been induced tumours. |
| Koletsky and Gustafson | 1955 | Rat (Wistar) | M (2 cases) | — | 1 out of 123 irradiated rats ; 1 out of 36 control rats. |
| Rosen, Castanera, Jones and Kimeldorf | 1961 | Rat (Sprague-Dawley) | M (2 cases) | — | 2 out of 141 irradiated rats. |
| Rowlatt | 1966 | Rat (Chester Beatty) | M (4 cases) | 16–25 m. | Multiple or solitary adenomas. 1026 males at risk (0·39%). |
| | | | F | 23 m. | Multiple adenomas. 226 females at risk (0·44%). |
| Rowlatt | 1966 | Mouse (C–) | F | 10 m. | Cystadenoma ; about 8500 mice at risk (0·01%). |
| *Carnivores* | | | | | |
| Schlegel | 1920 | St. Bernard dog | M | 6 | Multiple adenomas. May have been hyperplastic nodules. |
| Lombard | 1936 | Cocker spaniel | F | 9 | Multiple adenomas, possibly nodular hyperplasia. |
| Schlotthauer and Millar | 1955 | Dog | — (9 cases) | — | From a survey of 1150 tumours in 1024 dogs (0·78%). 6 tumours "of ductal origin". |

TABLE I.—*contd.*

| Author | Year | Animal | Sex | Age in years | Remarks |
|---|---|---|---|---|---|
| Garlt and Rössger | 1960 | Dog | — | — | From a survey of 143 tumours in 1788 dogs (0·7%). |
| Kronberger | 1960 | Dog | — (2 cases) | — | From a survey of 743 tumours in 9538 dogs (0·27%). |
| Thrasher | 1961 | German shepherd | M | 13 | From a survey of 145 tumours in 127 dogs (0·69%). |
| De Kock | 1962 | Scottie | M | 12 | Called "adenoma"; may have been of islet cell origin. |
| Fox | 1923 | Corsac fox (*Canis corsac*) | M | — | Adenoma of ductal origin. |
| | | Raccoon (*Procyon lotor*) | F | — | Two, separate, firm, encapsulated nodules. |
| Chesterman | 1965 | Ferret | F | 2¼ | Solitary adenoma. |
| Fox | 1933 | Mongoose (*Herpestes cafra*) | M | — | Multiple adenomas. |
| Hjärre | 1927 | Cat | — | — | One isolated and unusually large nodule in pancreas with nodular hyperplasia. |
| Lombard | 1936 | Cat | F | 13 | Multiple nodules; possibly hyperplastic. |
| Lechner | 1958 | Cat | — | — | From a survey of 32 tumours in 757 cats (3·1%). |
| *Artiodactyls* | | | | | |
| Kurtwig | 1910 | Ox | — | — | Pancreas weighed 45 kg.; adenoma weighed 190 g. |
| Boyd, Fitch, Grinnell and Billings | 1919 | Ox | F | — | Multiple encapsulated adenomas; may have been hyperplastic nodules. |
| Schlegel | 1920 | Ox | — (7 cases) | — | Cases seen during 15 year survey of tumours in cows. Pancreas weighed 500–700 g. |
| Kronberger | 1961 | Ox | — (6 cases) | — | From a survey of 738 tumours in cattle (0·81%). |
| Brandly and Migaki | 1963 | Ox | — (2 cases) | — | From a survey of 737 tumours in cattle (0·27%). |
| Machado, Lamas da Silva, Curial, Trein, Saliba, Martins, Cavalcanti, Dos Santos, Tokarnia, Döbereiner, Faria, Novloski and Da Costa Pereira | 1963 | Ox | — | — | From a survey of 551 tumours in 5028 cattle. |

TABLE II.—*Reports of Exocrine Adenocarcinoma in Mammals*

| Author | Year | Animal | Sex | Age in years | Remarks | Metastases |
|---|---|---|---|---|---|---|
| *Primates* (other than man) | | | | | | |
| Ratcliffe | 1930 | Grivet monkey (*Cercopithecus sabeus*) | F | — | Head of pancreas. | |
| Fox | 1931 | Yellow baboon (*Papio cynocephalus*) | F | 18+ | Locally invasive; in body of pancreas. | |
| *Lagomorphs* | | | | | | |
| Petit | 1909 | Rabbit | — | — | In omental accessory pancreas surrounded by coccidial cysts. | |
| *Rodents* | | | | | | |
| Plimmer | 1915 | Marmot | — | — | Carcinoma. | |
| Poel and Yerganian | 1961 | Chinese hamster (*Cricetulus griseus*) | F | — | Cystadenocarcinoma. | |
| Ashbel | 1945 | Syrian hamster (*Mesocricetus auratus*) | — | — | Carcinoma; 1 out of 13 tumours in 1000 animals. | |
| Fortner | 1957 | Syrian hamster | F | 3 | Carcinoma. From a survey of 46 tumours in 301 hamsters. | |
| Fortner | 1961 | Syrian hamster | M | — | | |
| Kirkman | 1962 | Syrian hamster | — | — | | |
| Chesterman | 1965 | Syrian hamster | M | 14 m. | Moderately well-differentiated tumour. | Spleen |
| | | ,, ,, | M | 29 m. | Adenocarcinoma. | Mesentery. |
| | | ,, ,, | F | 15 m. | Poorly differentiated adenocarcinoma. | |
| | | ,, ,, | F | 16 m. | Large intra-abdominal tumour replacing pancreas. | |
| Loeb | 1902 | Rat (white: strain unspecified) | F | — | Carcinoma. | |
| Wilson, DeEds and Cox | 1941 | Rat (Slonaker) | F | — | Carcinoma. | |
| Scott, Bostick, Shimkin and Hamilton | 1949 | Rat (Curtis-Dunning) | — | — | Poorly differentiated carcinoma. | |
| Elson | 1952 | Rat (Albino) | M | 18 m. | Carcinoma; 1 out of 130 rats in amino-stilbene feeding experiment. | |
| Hodge, Maynard and Blanchet | 1952 | Rat | F | 2 | Carcinoma; 1 out of 200 rats treated with methoxychlor. | |

TABLE II.—*contd.*

| Author | Year | Animal | Sex | Age in years | Remarks | Metastasis |
|---|---|---|---|---|---|---|
| Walpole, Roberts, Rose, Hendry and Homer | 1954 | Rat (Wistar) | M | 1 | Anaplastic carcinoma; 1 out of 91 control animals. No pancreatic tumours in 446 test animals given ethyleneimine derivatives. | |
| Griem | 1957 | Rat | F | 18 m. | Case report. Carcinoma. | Local nodes, peritoneum, omentum, liver and one intrathoracic deposit. |
| Gilbert and Gillman | 1958 | Rat (Wistar) | M | — | In a survey of 1114 tumours in 1342 untreated rats (0·17%). | |
| Ambrose, Booth, DeEds and Cox | 1960 | Rat (Albino) | F | — | Carcinoma; 1 out of 240 rats in non-tumour producing experiment. This animal in control group. | |
| Rosen, Castanera, Jones and Kimeldorf | 1961 | Rat (Sprague-Dawley) | M | — | 3 out of 141 rats in irradiation experiment, all in treated group. | |
| Snell | 1965 | Rat (M 520) | F | 30 m. | | |
| Rowlatt | 1966 | Rat (Chester Beatty) | M (2 cases) | 17 and 18 m. | 1026 male rats at risk (0·19%). | |
| | | | F | 2 | 226 female rats at risk (0·44%). | |
| Slye, Holmes and Wells | 1935 | White foot mouse (*Peromyscus novoboriensis*) | F | 2 | Pancreas and duodenum fused into solid mass. | Local lymph nodes. |
| | | | 2 | | | |
| | | Mouse (Albino) | M | 1 | Soft mass replacing pancreas. | Deposit attached to duodenum near pylorus. |
| Cloudman | 1941 | Mouse | — (3 cases) | — | In a survey of 20,000 tumours in mice (0·015%). | |
| Belkin | 1942 | Mouse (Bagg: albino) | — | — | 1 out of 800 mice injected with benzopyrene subcutaneously. | |
| Guérin | 1954 | Mouse | F | 2 | Firm tumour in mid-pancreas. | Right kidney and peritoneum. |
| Rowlatt | 1966 | Mouse (ICI strain) | F | 16½ m. | Moderately well differentiated adenocarcinoma. | Numerous peritoneal nodules. |

TABLE II.—*contd.*

| Author | Year | Animal | Sex | Age in years | Remarks | Metastasis |
|---|---|---|---|---|---|---|
| *Carnivores* | | | | | | |
| Nocard | 1877 | Spaniel | F | 6–7 | Large tumour in head of pancreas attached to local organs. | Liver. |
| Kitt | 1901 | Dog | — | — | Scirrhous carcinoma. | |
| Stephan | 1909 | Terrier | F | 14 | Hazel nut sized carcinoma. | |
| Ball | 1914 | Poodle | M | 9 | Acinar pancreatic tumour called "cancer". Also metastasizing sarcoma of small intestine. | None from pancreatic tumour. |
| Nieder | 1927 | Police dog | M | 8 | Pancreas weighed 1·6 kg. Replaced by locally invasive tumour. No microscopy. | |
| Bück | 1930 | Fox terrier | M | 10 | Early adenocarcinoma in head of pancreas. | |
| Günzel | 1931 | German sheep dog | F | — | Huge carcinoma. | Liver, lungs, spleen, lymph nodes, peritoneum. |
| Rudduck and Willis | 1938 | ,, ,, ,, | M | — | Tail of pancreas. | Spleen. |
| | | Collie | M | Adult | Large tumour in pancreas. | Numerous intraperitoneal and right transdiaphragmatic pleural deposits. |
| Harbitz | 1942 | Dog | — | 6 | Large hard mass invading duodenum. | Liver, lungs, portal and bronchial lymph nodes. |
| | | ,, | — | 11 | Large knobbly mass involving duodenum and constricting bile duct. | Liver, lungs, omentum, mesentery and portal lymph nodes. |
| Oliver | 1947 | Spaniel | F Spayed | Old | Obstructive mass in head of pancreas. | Local lymph nodes and wall of duodenum. |
| Stünzi | 1947 | Dog | (4 cases) | — | From survey of 304 carcinoma in 6325 dogs (1·32%). | |
| Krook | 1954 | Dog | — (9 cases) | — | From a survey of 440 tumours in 7248 dogs (2·05%). | |
| Schlotthauer and Millar | 1955 | Dog | — (7 cases) | — | From a survey of 521 carcinoma in 1150 dogs (1·34%). | |

TABLE II.—*contd.*

| Author | Year | Animal | Sex | Age in years | Remarks | Metastases |
|---|---|---|---|---|---|---|
| Plummer | 1956 | Dog | — | — | Tumour believed to have arisen in pancreas. | Spleen. |
| Gans | 1958 | Cocker spaniel | F | 10 | Large mass constricting duodenum and bile duct. | Liver. |
| Stünzi and Suter | 1958 | Spaniel | M | 9 | Head of pancreas. | Liver. |
| | | Niederlauf (short legged dog) | M | 8 | Head of pancreas. | Liver and omentum. |
| | | Boxer | M | 9 | Head of pancreas. | Liver, omentum, parietal peritoneum and spleen. |
| Cotchin | 1959 | Dog | — (15 cases) | — | From a survey of 4187 tumours in dogs (0·36%). | |
| Kronberger | 1960 | Dog | — (4 cases) | — | From a survey of 743 tumours in 9538 dogs (0·54%). | |
| Nobel and Neumann | 1960 | Dog | — | — | Adenocarcinoma. | |
| Überreiter | 1960 | Dog | — (4 cases) | — | From a survey of 4235 tumours (0·09%). | |
| Ditchfield and Archibald | 1961 | Scotch terrier | M | 9 | Tumour in body of pancreas. | Liver and lung. |
| Kadziolka | 1961 | Scotch terrier | M | 8 | Mass in head of pancreas. | Liver and lymph nodes. |
| Thrasher | 1961 | Dog | — | — | Adenocarcinoma. | |
| Cello and Olander | 1963 | Samoyed | F | 7 | Tumour in head of pancreas: thrombosis in portal vein and dural sinuses. | Liver, lung, ovaries, adrenal medulla, spleen, kidney, uterus, peritoneum, ribs, vertebra. |
| Machado *et al.* | 1963 | Dog | — | — | From a survey of 1956 tumours in 8760 dogs (0·05%). | |
| Mulligan | 1963 | Cocker spaniel | F | 5 | Adenocarcinoma. | Liver. |
| | | Cocker spaniel | F | 8 | ,, | ,, |
| | | Airedale terrier | F (spayed) | 3 | ,, | ,, |
| Sastry and Twiehaus | 1964 | Dog | F | 12 | Adenocarcinoma. | Lung and spleen. |
| | | Dog | — | Old | ,, | Liver. |
| | | Dog | — | Old | ,, From a survey of 291 neoplasms in dogs (1·03%). | Duodenum and portal lymph node. |

TABLE II.—*contd.*

| Author | Year | Animal | Sex | Ages in years | Remarks | Metastasis |
|---|---|---|---|---|---|---|
| Howard and Nielsen | 1965 | Boxers | — (4 cases) | — | Carcinoma. From a survey of 750 tumours in boxers and 750 tumours in other breeds. Incidence in both groups together = 0·53%. | |
| | | Other breeds of dog | — (4 cases) | — | | |
| Stünzi and Lott-Stolz | 1965 | Dog | (14 cases) | — | 1 poodle, 1 dachshund, 4 German sheepdogs, 5 spaniels, 3 boxers. From a survey of 746 tumours in 3787 dogs (1·88%). May include dogs reported by Stünzi and Suter (1958). | |
| Fox | 1931 | Jackal (*Canis anthus*) | F | — | Firm, greyish mass in head of pancreas. | Liver. |
| Kresky and Barnett | 1939 | Kodiak bear (*Ursus middendorfii*) | M | 31 | Stony hard mass in head of pancreas. | Local nodes and liver. |
| Goss | 1942 | Kodiak bear | F | 34 | Exactly similar tumour to above bear. | Local nodes and liver. |
| Ratcliffe | 1957 | Siberian brown bear (*Ursus arctos beringianus*) | M | 26 | Carcinoma. | |
| Chesterman and Pomerance | 1965 | Ferret | F | 2 | Adenocarcinoma. | |
| Fox | 1923 | Palm civet (*Paradoxurus niger*) | M | — | Head of pancreas twice normal size. | |
| Ball and Roquet | 1911 | Cat | F | 9 | Tail of pancreas. | Liver. |
| Mann and Brimhall | 1917 | Cat | M (Spayed) | 12 | Mass in head of pancreas. | Liver. |
| Scott and Moore | 1927 | Cat | M | Adult | Hard mass in head of pancreas. | Duodenum and retroperitoneal lymph nodes. |
| Bück | 1930 | Cat | M | 15 | Multiple neoplastic nodules mainly in head. | |
| D'Amore | 1948 | Cat | F | 12 | Head of pancreas. Poorly differentiated tumour; pancreatic origin inferred. | Liver, spleen, lungs, ovary, kidney, mesentery and local nodes. |

TABLE II.—*contd.*

| Author | Years | Animal | Sex | Age in years | Remarks | Metastasis |
|---|---|---|---|---|---|---|
| Mulligan | 1951 | Cat | — | — | Adenocarcinoma. From a survey of 66 tumours in cats (1·5%). | Liver. |
| Lechner | 1958 | Cat | — | — | From a survey of 32 tumours in 757 cats. | |
| Stünzi and Suter | 1958 | Cat | F | 16 | Cystic tumour in head of pancreas, probably from pancreatic duct. | None. |
| | | Cat | F | Old | Infiltrating, greyish white diffuse tumour. | Liver, lungs, mesenteric lymph nodes. |
| Cotchin | 1959 | Cat | — (9 cases ; 6 in spayed males) | — | From a survey of 571 tumours in cats (1·58%). | Metastases to liver in 6 cases. |
| Head | 1959 | Cat | — (11 cases) | — | From a survey of 413 tumours in cats (2·66%). | |
| Kronberger | 1960 | Cat | — (3 cases) | — | From a survey of 48 tumours in 2862 cats (6·25%). | |
| Urman and Tekeli | 1960 | Cat | F | 12 | Head of pancreas. | Liver, portal nodes, spleen and diaphragm. |
| Willis | 1960 | Cat | — (2 cases) | — | Adenocarcinoma. | |
| Ditchfield and Archibald | 1961 | Cat | M | 18 | Mass in head of pancreas. | |
| Machado *et al.* | 1963 | Cat | — (2 cases) | — | Called " carcinoma " and " adenocarcinoma ". From a survey of 93 tumours in 974 cats (2·15%). | |
| *Perissodactyls* (Odd-toed ungulates) | | | | | | |
| Martens | 1887 | Horse | — | — | Colloid carcinoma in head of pancreas. | Spleen. |
| Quentin | 1919 | Horse | M | 15 | Pancreas replaced by tumour. | Liver, lung. Tumour had invaded and perforated into a gastric diverticulum. |
| Balozet and Chainet | 1937 | Horse | M | 15 | 3·75 kg. irregular mass replacing pancreas. | |

TABLE II.—*contd.*

| Author | Year | Animal | Sex | Age in years | Remarks | Metastasis |
|---|---|---|---|---|---|---|
| *Artiodactyls* (Even-toed ungulates) | | | | | | |
| Machado *et al.* | 1963 | Pig | — | — | Adenocarcinoma. In a survey of 29 tumours in 2666 pigs. | |
| Ratcliffe | 1964 | Bactrian camel (*Camelus bactrianus*) | F | 26 | Carcinoma. | |
| Schlegel | 1920 | Ox | F | Old | Carcinoma weighing 570 g. | Invading portal vein. |
| Joest | 1912 | Ox | — | — | Carcinoma. | |
| Salomon | 1933 | Ox | F | Old | Partly encapsulated mass in pancreas. | |
| Marcato | 1942 | Ox | — | — | Epithelioma from exocrine pancreas. | |
| Jiřina | 1957 | Ox | F | 7 | Pancreas enlarged, firm, distorted. | Widespread, particularly to liver. |
| Ceretto | 1960 | Ox | — | 7 | Alveolar carcinoma. | Kidney. |
| Kronberger | 1961 | Ox | — | — | From a survey of 738 tumours in cattle (0·135%). | |
| Machado *et al.* | 1963 | Ox | — | — | In a survey of 551 tumours in 5028 oxen (0·18%). | |
| Jackson | 1936 | Sheep | F | Adult | Encapsulated tumour. | Liver, lung and probably spleen. |

TABLE III.—*Reports of Non-Metastasizing Islet Cell Tumours in Mammals*

| Author | Year | Animal | Sex | Age in years | Remarks |
|---|---|---|---|---|---|
| *Rodents* | | | | | |
| Fortner | 1961 | Syrian hamster (*Mesocricetus auratus*) | M (3 cases) | — | Adenoma in 4 out of 181 untreated animals. |
| | | | F (1 case) | — | |
| | | | M (2 cases) | — | Carcinoma in 2 out of 181 untreated animals. No metastasis mentioned. (Incidence of islet cell tumours = 3·3%.) |
| Dunham and Herrold | 1962 | Syrian hamster | — | — | Occasional (1–14 animals) adenomas in 360 old hamsters used in a negative experiment. |
| Kirkman | 1962 | Syrian hamster | — (3 cases) | — | 3 out of 7200 animals. Also 1 adenocarcinoma of islets but no metastases mentioned (0·05%). |
| Chesterman | 1965 | Syrian hamster | F | 17 m. | Untreated. |
| Benitz and Kramer | 1965 | Mongolian gerbil (*Meriones unguiculatus*) | M | 2 | From a survey of 25 tumours in 106 |
| | | | M | 2 | gerbils (8·0%). |
| Bagg and Hagopian | 1939 | Rat (Wistar) | F | 26 m. | 92 rats at risk in rapid breeding experiment without suckling. |
| Guérin | 1954 | Rat | M | 2 | Castrated aged 2 months. Later grafted with his mother's ovary. |
| Walpole, Roberts, Rose, Hendry and Homer | 1954 | Rat (Wistar) | M | 22 m. | 1 out of 91 control animals. 446 test animals without pancreatic tumour. |
| Hueper | 1955*a* | Rat (Wistar) | — | — | 1 out of 100 rats given intrafemoral nickel. |
| | 1955*b* | Rat (Wistar) | M | — | 1 out of 25 rats given intrafemoral chromium. |
| | | | M (2 cases) | — | 2 out of 25 rats given intraperitoneal chromium. |
| Koletsky and Gustafson | 1955 | Rat (Wistar) | M (9 cases) | — | 12 islet cell tumours in 8 out of 123 irradiated rats. One other rat had a carcinoma of islets without metastasis. |
| Ives and Dack | 1957 | Rat (Sprague-Dawley) | — (3 cases) | — | 3 out of 540 rats in negative experiment. 224 tumours mostly in control group. Incidence of islet cell tumours = 1·34%. |
| Gilbert and Gillman | 1958 | Rat (Wistar) | M (17 cases) F (6 cases) | — | 1342 untreated rats with 1114 tumours. 16 adenomas (2·77% all tumours in males) and 1 carcinoma with metastasis in males. 6 adenomas (1·12% all tumours in females); no carcinoma in females. |

TABLE III.—*contd.*

| Author | Year | Animal | Sex | Age in years | Remarks |
|---|---|---|---|---|---|
| Berdjis | 1960 | Rat (Sprague-Dawley) | — (3 cases) | — | Multiple adenomas, one associated with a papillary cystadenoma of the pancreas of uncertain nature, in 3 irradiated rats. |
| Jasmin | 1961 | Rat (Sprague-Dawley) | — | — | 1 out of 100 rats; 80 given carboxy-methyl-cellulose including this animal. |
| Rosen, Castanera, Jones and Kimeldorf | 1961 | Rat (Sprague-Dawley) | M (22 cases) | — | 22 out of 141 rats (15·6%) in irradiation experiment, 18% in treated group, 9·8% in control group. |
| Thompson, Huseby, Fox, Davis and Hunt | 1961 | Rat (Sprague-Dawley) | — | — | 1 out of 125 untreated rats. |
| Morris, Wagner, Ray, Stewart and Snell | 1962 | Rat (Buffalo) | M | — | Papillary adenoma in 1 out of 79 rats. 59 given N,N′-2,7-fluorenylenebis-acetamide including this animal. |
| Rosen, Castanera, Kimeldorf and Jones | 1962 | Rat (Sprague-Dawley) | M | — | Multiple adenomas of islets in 49% of 115 irradiated rats and in 17% of control animals. |
| Barron | 1963 | Rat (Charles River) | M | — | 1 out of 90 rats; 60 treated with 3-phenyl-5 β-diethylaminoethyl-1,2,4-oxadiazole including this animal. |
| Berdjis | 1963 | Rat (Sprague-Dawley) | — (10 cases) | — | Islet cell tumours in 8 out of 37 irradiated rats (21·6%) and in 2 out of 36 controls (5·4%). |
| Warren, Carlstein, Steinke and Chute | 1964 | Rat (Deac-Slonaker | Parabients | — | 715 pairs of irradiated parabiotic rats with 113 control pairs. 40 adenomas in irradiated partner, 2 adenomas in non-irradiated animal. 1 adenoma control group. |
| Snell | 1965 | Rat (WN) | M | 20 m. | Adenoma |
| | | (ACI) | F | 21 m. | Adenoma |
| Baker and Tucker (personal communication) | 1966 | Rat (Wistar ICI) | M (9 cases) | 29–38 m. | Adenoma in 6 male rats and non-metastasizing carcinoma in 5 (3 male, 2 female) out of 1200 untreated rats. |
| | | | F (2 cases) | 29 and 32 m. | |
| Rowlatt | 1966 | Rat (Chester Beatty) | M (2 cases) | 20 and 25 m. | 2 out of 1252 rats at risk (0·16%). |
| Hueper | 1936 | White mouse | — | — | Incidental finding in mouse exposed to carbon disulphide vapour for 4 months. |

TABLE III.—*contd.*

| Author | Year | Animal | Sex | Age in years | Remarks |
|---|---|---|---|---|---|
| Bielschowsky, Bielschowsky and Lindsay | 1956 | Mouse (NZY) | F | — | 1 out of 204 mice at risk. |
| Dunn and Green | 1963 | Mouse (BALB/c) | M | — | 1 out of 51 mice at risk. |
| Hansen | 1949 | St. Bernard dog | F | 8 | Solitary nodule; tumour invading capsule only. No hypoglycaemia. |
| Schlotthauer and Millar | 1955 | Dog | — | — | From a survey of 1150 tumours in 1024 dogs (0·09%). |
| Hansen and Krock | 1958 | Fox terrier | — | 9 | Solitary nodule in duodenal portion. Hypoglycaemia. |
| | | Boxer | — | 10 | Two nodules, in angle and duodenal portion. Hypoglycaemia. |
| | | Collie | — | 7 | Solitary nodule in angle. Hypoglycaemia. |
| Grant | 1960 | Boxer | F | 8 | Solitary; capsule thin but not involved. |
| Runnels, Monlux and Monlux (for E. L. Stubbs) | 1960 | Mongrel | — | 12 | Solitary nodule. Hypoglycaemia relieved by excision. |
| Teunissen, Verwer and Van Den Akker | 1961 | Airedale terrier | F | 10 | Two tumours in duodenal portion, one penetrating the capsule. |
| Thrasher | 1961 | Mongrel | F | 12 | Islet cell tumours (one was a carcinoma without metastasis) from a survey of |
| | | Wire-haired terrier | M | 4 | 145 tumours in 127 dogs (1·4%). |
| Justus | 1963 | Boxer | M | 8½ | Solitary nodule. Hypoglycaemia relieved by partial pancreatectomy. |
| Marder | 1963 | Irish setter | F | 9 | Solitary nodule. Hypoglycaemia. |
| Marcus, Bucci and Kraner | 1964 | Fox terrier | F | 12 | Solitary, encapsulated tumour invad-capsule only. Hypoglycaemia. |
| Wilkinson | 1964 | Boxer | F | 6 | Solitary nodule in duodenal portion infiltrating locally only. Hypoglycaemia relieved by local excision. |
| Howard and Nielsen | 1965 | Boxer | M | 6 | 2 out of 750 Boxers. Both dogs also had aortic body tumours and |
| | | Boxer | — | — | multiple endocrine tumours. |
| Ball | 1924 | Cat | — | — | Epithelioma. No metastases described. |
| Smith and Jones (for M. A. Troy) | 1961 | Persian cat | F | 10 | Photomicrograph but no details. |
| *Artiodactyls* | | | | | |
| Tokarnia | 1961 | Ox | F | 10 | Multiple nodules. Tumour involving capsule only. |
| Asdrubali, Latini and Patrizi | 1964 | Ox | F | 10 | Solitary nodule. Rather doubtful history of hypoglycaemia. |

TABLE IV.—*Reports of Metastasizing Islet Cell Tumours in Mammals*

| Author | Year | Animal | Sex | Age in years | Remarks | Metastasis |
|---|---|---|---|---|---|---|
| *Rodents* | | | | | | |
| Chesterman | 1965 | Syrian hamster (*Mesocricetus auratus*) | M | 23½m. | Carcinoma. Controversial. May have been of exocrine origin. | Liver. |
| | | Syrian hamster | M | 22m. | Tumour replacing pancreas. May have been of exocrine origin. | Liver. |
| *Carnivores* | | | | | | |
| Bru | 1927 | Dog | — | — | Multiple nodules. | Lung, heart, liver. bronchial lymph nodes. |
| Slye and Wells | 1935 | Bull terrier | F | 12½ | Multiple adenomas at least two of which were carcinomas. Hypoglycaemia. | Local lymph node. |
| Hansen | 1949 | German boxer | M | 8 | Solitary tumour. | Liver. |
| Cello and Kennedy | 1957 | Cocker spaniel | M | 6½ | Multiple tumours. Hypoglycaemia. | Liver and nodes. |
| | | Boxer | M | 5 | Solitary nodule. Hypoglycaemia. | Liver and nodes. |
| | | Red-bone hound | — | 10 | Solitary nodule. Hypoglycaemia. | Local nodes. |
| Hansen and Krook | 1958 | Doberman Pinscher | — | 10 | Solitary nodule in splenic part. Hypoglycaemia. | Liver ; duodenal and portal lymph nodes. |
| Hansen (quoted by Grant, 1960) | 1960 | Dog | — (2 cases) | — | Solitary tumours. | Local lymph nodes and liver. |
| Krook and Kenney | 1962 | Boxer | F | 10 | Solitary nodule. Hypoglycaemia. | Portal lymph nodes. |
| | | Bosian terrier. | M | 6 | Solitary nodule. Hypoglycaemia. | Local lymph nodes and liver. |
| Beck and Krook | 1965 | Fox terrier | M | 9 | Large tumour in head of pancreas. Hypoglycaemia relieved temporarily by operation. | Liver. Local lymph nodes. |
| | | Cocker spaniel | F | 5 | Tumour in splenic portion of pancreas. | Liver. Portal lymph node. |
| Lombard | 1935 | Cat | M | 3 | Tail of pancreas. | Liver. |

TABLE IV.—*contd.*

| Author | Year | Animal | Sex | Age in years | Remarks | Metastasis |
|---|---|---|---|---|---|---|
| *Artiodactyls* | | | | | | |
| Montroni | 1932 | Ox | — | — | Multiple scattered nodules. | Kidney. |
| Barile | 1936 | Ox | — | 4 | Head of pancreas, attached to hilum of liver. | Liver and local lymph nodes. |
| Marcato | 1942 | Ox | — (9 cases) | — | Islet cell " blastomas ". Composite description of cases. | Metastasis said to be frequent. |
| Montroni | 1957 | Ox | — | — | Poorly differentiated tumour probably from pancreas. | Right diaphragmatic peritoneum. |
| Montroni | 1958 | Ox | — | — | Solitary nodule. | Intrahepatic vena cava. |
| Asdrubali, Latini and Patrizi | 1964 | Ox | F | 8 | Numerous nodules. | Pancreatic and portal lymph nodes, liver, diaphragmatic and parietal peritoneum. |
| | | Ox | F | 10 | Nodular pancreas. " Nervous attacks " | Liver. |
| | | Ox | F | 8 | Numerous nodules. | Pancreatic and portal lymph nodes, portal vein and liver. |

SUMMARY

Reports of spontaneously occurring benign and malignant epithelial tumours of exocrine and islet origin in mammals have been arranged in four tables according to histological appearances. This review shows that adenocarcinoma is commoner in man, and perhaps in the dog, than in other mammalian species. However, adenocarcinoma of the pancreas has been described fairly often in several unrelated mammalian orders and should be regarded as a neoplasm which affects a wide range of animal species. A plea is made for better standards of description of pancreatic tumours and a larger and more comprehensive sample derived from animals at present available for study but often examined only cursorily.

REFERENCES

AMBROSE, A. M., BOOTH, A. N., DEEDS, F. AND COX, A. J.—(1960) *Fd Res.*, **25**, 328.
ASDRUBALI, G., LATINI, A. AND PATRIZI, R.—(1964) *Archo vet. ital.*, **15**, 191.
ASHBEL, R.—(1945) *Nature, Lond.*, **155**, 607.
BAGG, H. J. AND HAGOPIAN, F.—(1939) *Am. J. Cancer*, **35**, 175.
BALL, V.—(1914) *J. méd. vét. Zootech.*, **65**, 391.—(1924) ' Traité d'anatomie pathologique générale '. Paris (Vigot Frères), pp. 455–458.
BALL, V. AND ROQUET,—(1911) *J. méd. vét. Zootech* **62**, 477.
BALOZET, L. AND CHAINET, L.—(1937) *Bull. Acad. vét. Fr.*, **10**, 301.
BARILE, C.—(1936) *Nuovo Ercol.*, **41**, 153.
BARRON, C. N.—(1963) *Exp. Molec. Path.*, Suppl., **2**, 1.
BASHFORD, E. F. AND MURRAY, J. A.—(1904) *Rep. imp. Cancer Res. Fund*, **1**, 3.
BAUMGÄRTNER, H.—(1929) *Z. Krebsforsch.*, **28**, 241.
BECK, A. M. AND KROOK, L.—(1965) *Cornell Vet.*, **55**, 330.
BELKIN, M.—(1942) *Cancer Res.*, **2**, 264.
BELL, E. T.—(1957) *Am. J. Path.*, **33**, 499.
BENITZ, K-F. AND KRAMER, A. W.—(1965) *Lab. Anim. Care*, **15**, 281.
BERDJIS, C. C.—(1960) *Oncologia, Basel*, **13**, 441.—(1963*a*) *Exp. Molec. Path.*, **2**, 157.—(1963*b*) *Oncologia, Basel*, **16**, 81.
BIELSCHOWSKY, M., BIELSCHOWSKY, F. AND LINDSAY, D.—(1956) *Br. J. Cancer*, **10**, 688.
BLUMENTHAL, H. T. AND ROGERS, J. B.—(1965) ' Spontaneous and induced tumors in the guinea-pig '. In ' The pathology of laboratory animals ', edited by Ribelin, W. E. and McCoy, J. R. Springfield, Illinois (Charles C. Thomas).
BOYD, W. L., FITCH, C. P., GRINNELLS, C. D. AND BILLINGS, W. A.—(1919) *Cornell Vet.*, **9**, 169.
BRANDLY, P. J. AND MIGAKI, G.—(1963) *Ann. N.Y. Acad. Sci.*, **108**, 872.
BRU, P.—(1927) *Revue méd.-chir. Mal. Foie Pancr. Rate*, **2**, 40.
BÜCK, G.—(1930) ' Contribution à l'Etude des tumeurs du Pancréas chez les animaux '. (Thesis.) Lyon.
CELLO, R. M. AND KENNEDY, P. C.—(1957) *Cornell Vet.*, **47**, 538.
CELLO, R. M. AND OLANDER, H.—(1963) *J. Am. vet. med. Ass.*, **142**, 1407.
CERETTO, F.—(1960) *Annali Fac. Med. vet. Torino*, **10**, 37.
CHESTERMAN, F. C. AND POMERANCE, A.—(1965) *J. Path. Bact.*, **89**, 529.
CLOUDMAN, A. M.—(1941) ' Spontaneous neoplasms in mice. In Biology of the laboratory mouse ', edited by SNELL, G. D. Philadelphia (Blakiston Company).
COHRS, P., JAFFÉ, R. AND MEESSEN, H.—(1958) ' Pathologie der laboratoriumstiere '. 2 vols. Berlin (Springer).
COTCHIN, E.—(1956) ' Neoplasms of the domesticated mammals '. Farnham Royal, England (Commonwealth Agricultural Bureaux).—(1959) *Vet. Rec.*, **71**, 1040.—(1962) *Bull. Wld Hlth Org.*, **26**, 633.
D'AMORE, A.—(1948) *Rc. Ist. sup. Sanità*, **11**, 557.

De Kock, G.—(1962) *Onderstepoort J. vet. Res.*, **29**, 35.
Ditchfield, J. and Archibald, J.—(1961) *Small Anim. Clin.*, **1**, 173.
Dobberstein, J., Pallaske, G. and Stünzi, H.—(1963) ' Handbuch der speziellen pathologischen Anatomie der Haustiere '. 3rd auf Band VI. Berlin (Parey), pp. 331–335.
Dorn, C. R.—(1964) *Nature, Lond.*, **202**, 513.
Dunham, L. J. and Herrold, K. M.—(1962) *J. natn. Cancer Inst.*, **29**, 1047.
Dunn, T. B. and Green, A. W.—(1963) *J. natn. Cancer Inst.*, **31**, 425.
Eichler.—(1901) *Z. Tiermed.*, **5**, 428.
Elson, L. A.—(1952) *Br. J. Cancer*, **6**, 392.
Fardeau, G.—(1931) ' Les tumeurs spontanées chez le lapin. Revue critique '. Thesis, Paris, p. 46.
Feldman, W. H.—(1932) ' Neoplasms of domesticated animals '. Philadelphia (W. B. Saunders).
Fischer, W. and Kühl, I.—(1958) ' Geschwülste der Laboratoriums-nagetiere '. Dresden and Leipzig (Theodor Steinkopff).
Fortner, J. G.—(1957) *Cancer, N.Y.*, **10**, 1153.—(1961) *Cancer Res.*, **21**, 1491.
Fox, H.—(1923) ' Disease in captive wild mammals and birds '. Philadelphia, London and Chicago (Lippincott), pp. 227 and 259.—(1931) *Rep. Lab. Mus. comp. Path. zool. Soc. Philad.*, pp. 22 and 23.—(1933) *Rep. Lab. Mus. comp. Path. zool. Soc. Philad.*, p. 19.
Frantz, V. K.—(1959) ' Tumors of the pancreas. Atlas of tumor pathology '. Section 7, fasc. 27 and 28. Washington, D.C. (Armed Forces Institute of Pathology).
Gamgée.—(1856) ' Tumeurs multiples chez la jument. The Veterinarian ', p. 203. Cited by Bück, G. (1930) (see above).
Gans, J. H.—(1958) *Cornell Vet.*, **48**, 372.
Garlt, C. and Rössger, M.—(1960) *Mh. VetMed.*, **15**, 153.
Gilbert, C. and Gillman, J.—(1958) *S. Afr. J. med. Sci.*, **23**, 257.
Glenner, G. G. and Mallory, G. K.—(1956) *Cancer, N.Y.*, **9**, 980.
Goss, L. J.—(1942) *J. tech. Meth. Bull. int. Ass. med. Mus.*, **22**, 27.
Grant, C. A.—(1960) *J. comp. Path. Ther.*, **70**, 450.
Griem, W.—(1957) *Berl. Münch. tierärztl. Wschr.*, **70**, 451.
Guérin, M.—(1954) ' Tumeurs spontanées des animaux de laboratoire '. Paris (Amédée Legrand), pp. 124 and 125.
Günzel, F.—(1931) ' Ueber zwei Fälle von primärem Carcinom des Pancreas bei Hunden '. Inaug-dissert. Hannover.
Hansen, H.-J.—(1949) *Nord. VetMed.*, **1**, 363.
Hansen, H-J. and Krook, L.—(1958) VIII Nordiska veterinärmötet-Sektion F, Rapport 2. Helsinki, p. 886.
Harbitz, F.—(1942) *Norsk. VetTidsskr.*, **54**, 193.
Head, K. W.—(1959) *Vet. Rec.*, **71**, 1052.
Hendry, J. A., Matthews, J. J., Walpole, A. L. and Williams, M. H. C.—(1955) *Nature, Lond.*, **175**, 1131.
Hjärre, A.—(1927) *Arch. wiss. prakt. Tierheilk.*, **57**, 1.
Hodge, H. C., Maynard, E. A. and Blanchet, H. J. Jr.—(1952) *J. Pharmac. exp. Ther.*, **104**, 60.
Howard, E. B. and Nielsen, S. W.—(1965) *Am. J. vet. Res.*, **26**, 1121.
Hueper, W. C.—(1936) *Archs Path.*, **22**, 220.—(1955*a*) *J. natn. Cancer Inst.*, **16**, 55.—(1955*b*) *J. natn. Cancer Inst.*, **16**, 447.
Innes, J. R. M.—(1958) ' Malignant diseases in domesticated animals '. In ' Cancer ', edited by Raven, R. W. London (Butterworth). Vol. 3, pp. 73–115.
Ives, M. and Dack, G. M.—(1957) *Fd. Res.*, **22**, 102.
Jackson, C.—(1936) *Onderstepoort J. vet. Sci. Anim. Ind.*, **6**, 3.
Jasmin, G.—(1961) *Revue can. Biol.*, **20**, 701.
Jiřina, K.—(1957) *Berl. Münch. tierärztl. Wschr.*, **70**, 490.

JOEST.—(1912) *Dresdener tierärztliche Hochschule Ber.*, **7**, 101. Cited by Slye, M., Holmes, H. F. and Wells, H. G. (1935) (see below).
JUNGHERR, E.—(1963) *Ann. N.Y. Acad. Sci.*, **108**, 777.
JUSTUS, H. A.—(1963) *J. Am. vet. med. Ass.*, **142**, 1413.
KADZIOLKA, A.—(1961) *Medycyna wet.*, **17**, 205.
KIRKMAN, H.—(1962) *Stanford med. Bull.*, **20**, 163.
KITT.—(1901) *Lehrb. d. path. Anat. d. Haustiere*, p. 600. Cited by Sticker, A.,—(1920) *Arch. klin. Chir.*, **65**, 616.
KOLETSKY, S. AND GUSTAFSON, G. E.—(1955) *Cancer Res.*, **15**, 100.
KRESKY, P. J. AND BARNETT, R. N.—(1939) *Zoologica, N.Y.*, **24**, 285.
KRONBERGER, H.—(1960) *Mh. VetMed.*, **15**, 730.—(1961) *Mh. VetMed.*, **16**, 296.
KROOK, L.—(1954) *Acta path. microbiol. scand.*, **35**, 407.
KROOK, L. AND KENNEY, R. M.—(1962) *Cornell Vet.*, **52**, 385.
KURTWIG.—(1910) ' Pankreasadenom bei einer Kuh '. Jber. beamt. Tierarzte Preussens 2, teil 51. Cited by Dobberstein, J., Pallaske, G. and Stünzi, H. (1963) (see above).
LECHNER, M.—(1958) ' Spontantumoren bei Säugetieren. Ein Beitrag zur vergleichenden Geschwülstforschung '. Inaug-dissert. München.
LIÉNAUX.—(1895) *Ann. Méd. vét.*, **44**, 360.
LOEB, L.—(1902) *J. med. Res.*, **8**, 44.
LOMBARD, C.—(1935) *Revue vét., Toulouse*, **87**, 632.—(1936) *Revue vét., Toulouse*, **88**, 672.—(1962) ' Cancérologie comparée '. Paris (G. Doin).
LOMBARD, L. S. AND WITTE, E. J.—(1959) *Cancer Res.*, **19**, 127.
MCDIARMID, A.—(1962) *F.A.O. agric. Stud.*, **57**, 1.
MACHADO, A. V., LAMAS DA SILVA, J. M., CURIAL, O., TREIN, E. J., SALIBA, A. M., MARTINS, E. O., CAVALCANTI, M. I., DOS SANTOS, J. A., TOKARNIA, C. H., DÖBEREINER, J., FARIA, J. F., NOVLOSKI, G. AND DA COSTA PEREIRA, E. F.—(1963) *Escola de veterinaria*, **15**, 327.
MANN, F. C. AND BRIMHALL, S. D.—(1917) *J. Am. vet. med. Ass.*, **52**, 195.
MARCATO, A.—(1942) *Nuova Vet.*, **21**, 3.
MARCUS, L. C., BUCCI, T. J. AND KRANER, K. L.—(1964) *J. Am. vet. med. Ass.*, **145**, 1198.
MARDER, M. W.—(1963) *The Veterinarian, Mass.*, **9**, 13.
MARTENS.—(1887) *Arch. wiss. prakt. Tierheilk.*, **13**, 368.
MESSNER, E.—(1909) *Dt. tierärztl. Wschr.*, **27**, 396.
MONTRONI, L.—(1932) *Nuova Vet.*, **10**, 315.—(1957) *Zooprofilassi*, **12**, 372.—(1958) *Zooprofilassi*, **13**, 451.
MORRIS, H. P., WAGNER, B. P., RAY, F. E., STEWART, H. L. AND SNELL, K. C.—(1962) *J. natn. Cancer Inst.*, **29**, 977.
MOULTON, J. E.—(1961) ' Tumors in domestic animals '. Berkeley and Los Angeles (Univ. of California Press), p. 139.
MULLIGAN, R. M.—(1951) *Cancer Res.*, **11**, 271.—(1963) *Ann. N.Y. Acad. Sci.*, **108**, 642.
NIEDER, M.—(1927) *Revue vét., Toulouse*, **79**, 683.
NOBEL, T. A. AND NEUMANN, F.—(1960) *Refuah vet.*, **17**, 39.
NOCARD, É.—(1877) *Arch. vét.*, **2**, 451.
OLIVER, P. A.—(1947) *Vet. Med.*, **42**, 267.
PETISCA, J. L. N.—(1947) *Repos. Trab. Lab. Patol. vet., Lisb.*, **6**, 361.
PETIT.—(1909) ' Carcinome développé dans un pancréas accessoire '. Ass. fr. du Cancer, pp. 25 and 28. Cited by Fardeau, G. (1931) (see above).
PLIMMER, H. G.—(1915) *Proc. zool. Soc. Lond.*, **1915**, 123.
PLUMMER, P. J. G.—(1956) *Can. J. comp. Med.*, **20**, 239.
POEL, W. E. AND YERGANIAN, G.—(1961) *Am. J. Med.*, **31**, 861.
QUENTIN.—(1919) *Bull. Mém. Soc. cent. Méd. vét.*, **72**, 290.
RATCLIFFE, H. L.—(1930) *J. Cancer Res.*, **14**, 453.—(1957) *Rep. Penrose Res. Lab. zool. Soc. Philad.*, p. 10.—(1964) *Rep. Penrose Res. Lab. zool. Soc. Philad.*, p. 14.
RIBELIN, W. E. AND MCCOY, J. R.—(1965) ' The pathology of laboratory animals '. Springfield, Illinois (Charles C. Thomas).

Rosen, V. J. Jr., Castanera, T. J., Jones, D. C. and Kimeldorf, D. J.—(1961) *Lab. Invest.*, **10**, 608.
Rosen, V. J. Jr., Castanera, T. J., Kimeldorf, D. J. and Jones, D. C.—(1962) *Lab. Invest.*, **11**, 204.
Rowlatt, U.—(1966) ' Spontaneous neoplasms of mouse and rat pancreas '. In ' The pathology of laboratory rats and mice'. Edited by Cotchin, E. and Roe, F. J. C. Oxford (Blackwell). In press.
Ruch, T. C.—(1959) ' Diseases of Laboratory Primates '. Philadelphia and London (W. B. Saunders and Co.).
Rudduck, H. B. and Willis, R. A.—(1938) *Am. J. Cancer*, **33**, 205.
Runnells, R. A., Monlux, W. S. and Monlux, A. W.—(1960) ' Principles of Veterinary Pathology '. Iowa State University, Ames, U.S.A. p. 517.
Salomon, S.—(1933) *Berl. tierärztl. Wschr.*, **15**, 230.
Sastry, G. A. and Twiehaus, M. J.—(1964) *Indian vet. J.*, **41**, 317.
Schlegel, M.—(1911) *Z. Tiermed.*, p. 223. Cited by Bück, G. (1930) (see above).—(1920) *Berl. tierärztl. Wschr.*, **36**, 529.
Schlotthauer, C. F. and Millar, J. A. S.—(1955) ' Tumors in dogs '. Papers of 5th Gaines vet. symposium entitled ' The newer knowledge about dogs ', pp. 21–24.
Scott, K. G., Bostick, W. L., Shimkin, M. B. and Hamilton, J. G.—(1949) *Cancer N.Y.*, **2**, 692.
Scott, E. and Moore, R. A.—(1927) *J. Cancer Res.*, **11**, 152.
Simpson, G. G.—(1945) *Bull. Am. Mus. nat. Hist.*, **85**, 34.
Slye, M., Holmes, H. F. and Wells, H. G.—(1935) *Am. J. Cancer*, **23**, 81.
Slye, M. and Wells, H. G.—(1935) *Archs Path.*, **19**, 537.
Smith, H. A. and Jones, T. C.—(1961) ' Veterinary pathology '. London (Henry Kimpton), pp. 190 and 247.
Snell, K. C.—(1965) ' Spontaneous lesions of the rat '. In ' The pathology of laboratory animals ', edited by Ribelin, W. E. and McCoy, J. R. Springfield, Illinois (Charles C. Thomas).
Snyder, R. L. and Ratcliffe, H. L.—(1963) *Ann. N.Y. Acad. Sci.*, **108**, 793.
Steiner, P. E. and Bengston, J. S.—(1951) *Cancer, N.Y.*, **4**, 1113.
Stephan.—(1909) ' Die tumoren in der Leber des Hundes '. Inaug-dissert., Giessen. Cited by Günzel, F. (1931) (see above).
Stünzi, H.—(1947) *Skand. VetTidskr.*, **37**, 453.
Stünzi, H. and Lott-Stolz, G.—(1965) *Mh. VetMed.*, **20**, 793.
Stünzi, H. and Suter, P.—(1958) *Mh. VetMed.*, **13**, 251.
Tamaschke, C.—(1951–52) *Wiss. Z. Humboldt-Univ. Berl.*, **1**, 37.—(1955) *Strahlentherapie*, **96**, 150.
Teunissen, G. H. B., Verwer, M. A. J. and Van Den Akker, S.—(1961) *Tijdschr. Diergeneesk.*, **86**, 1115.
Thompson, S. W., Huseby, R. A., Fox, M. A., Davis, C. L. and Hunt, R. D.—(1961) *J. natn. Cancer Inst.*, **27**, 1037.
Thrasher, J. P.—(1961) *J. Am. vet. Med. Ass.*, **138**, 27.
Tokarnia, C. H.—(1961) *J. Am. vet. Med. Ass.*, **138**, 541.
Überreiter, O.—(1960) *Wien. tierärztl. Mschr.*, **47**, 805.
Upton, A. C., Kimball, A. W., Furth, J., Christenberry, K. W. and Benedict, W. H.—(1960) *Cancer Res.*, **20**, (2), 1.
Urman, H. K. and Tekeli, S.—(1960) *Vet. Fak. Derg. Ankara Univ.*, **7**, 124.
Walpole, A. L., Roberts, D. C., Rose, F. L., Hendry, J. A. and Homer, R. F.—(1954) *Br. J. Pharmac. Chemother.*, **9**, 306.
Warren, S., Carlstein, R. G., Steinke, J. and Chute, R. N.—(1964) *Proc. Soc. exp. Biol. Med.*, **115**, 910.
Wilkinson, J. S.—(1964) *J. Am. vet. med. Ass.*, **144**, 404.
Willis, R. A.—(1960) ' Pathology of tumours '. Third edition. London (Butterworths), pp. 97 and 443.
Wilson, R. H., DeEds, F. and Cox, A. J.—(1941) *Cancer Res.*, **1**, 595.